# Tolerability of vortioxetine compared to selective serotonin reuptake inhibitors in older adults with major depressive disorder (VESPA): a randomised, assessor-blinded and statistician-blinded, multicentre, superiority trial

**DOI:** 10.1016/j.eclinm.2024.102491

**Published:** 2024-02-15

**Authors:** Giovanni Ostuzzi, Chiara Gastaldon, Mauro Tettamanti, Massimo Cartabia, Igor Monti, Andrea Aguglia, Eugenio Aguglia, Francesco Bartoli, Camilla Callegari, Andrea Canozzi, Elvira Anna Carbone, Giuseppe Carrà, Rosangela Caruso, Simone Cavallotti, Stefania Chiappini, Fabrizio Colasante, Beatrice Compri, Armando D'Agostino, Pasquale De Fazio, Renato de Filippis, Matteo Gari, Marta Ielmini, Gianmarco Ingrosso, Silvia Mammarella, Giovanni Martinotti, Alessandro Rodolico, Rita Roncone, Enrico Sterzi, Lorenzo Tarsitani, Elisa Tiberto, Liliana Todini, Francesco Amaddeo, Barbara D'Avanzo, The VESPA Study Group, Eugenio Aguglia, Andrea Aguglia, Maria Chiara Alessi, Gabriele Avincola, Bianca Bachi, Angelo Barbato, Corrado Barbui, Francesco Bartoli, Gianna Bernasconi, Andrea Birgillito, Emanuele Bisso, Stefano Bonora, Angela Calabrese, Camilla Callegari, Tommaso Callovini, Aurelia Canestro, Salvo Canonico, Chiara Alessandro Capogrosso, Elvira Carbone, Doriana Carosielli, Giuseppe Carrà, Massimo Cartabia, Ivano Caselli, Daniele Cavaleri, Simone Cavallotti, Clara Cavallotto, Marco Cesca, Cecilia Chiarenza, Riccardo Matteo Cioni, Sara Coloccini, Marco Cruciata, Claudia Cumerlato, Armando D'Agostino, Barbara D'Avanzo, Pasquale De Fazio, Renato De Filippis, Manuela De Palma, Sasha Del Vecchio, Bianca Della Rocca, Chiara Di Natale, Ettore D'Onofrio, Irene Espa, Giulia Fior, Marta Gancitano, Matteo Gari, Chiara Gastaldon, Barbara Giordano, Laura Giusti, Luigi Grassi, Pierluca Guzzi, Marta Ielmini, Gianmarco Ingrosso, Celeste Isella, Annamaria Lax, Silvia Mammarella, Leonardo Marano, Federico Marconi, Marco Marella, Alessia Metelli, Giulia Michencig, Andrea Miuli, Alessandro Moncada, Igor Monti, Pietro Morello, Federico Moretti, Marco Morreale, Alessio Mosca, Christian Nasti, Michela Nosé, Filippo Ogheri, Margherita Oresti, Alessandra Ornaghi, Giovanni Ostuzzi, Dario Palpella, Corinna Pancheri, Davide Papola, Silvia Passeri, Mauro Pettorusso, Susanna Piacenti, Irene Pinucci, Valentina Pugliese, Marianna Purgato, Marianna Rania, Federica Robbi, Alessandro Rodolico, Samantha Romito, Barbara Ronchi, Rita Roncone, Valentina Roselli, Cristina Segura-Garcia, Maria Salvina Signorelli, Gabriele Simonelli, Antonella Sociali, Enrico Sterzi, Serena Sturiale, Antonio Tambelli, Mauro Tettamanti, Beatrice Todesco, Alice Trabucco, Giulia Turrini, Veronica Villa, Federico Wiedenmann, Luca Zambuto, Elisa Zanini, Chiara Zannini, Luigi Zerbinati, Angelo Barbato, Corrado Barbui

**Affiliations:** a Department of Neuroscience, Biomedicine and Movement Sciences, Section of Psychiatry, University of Verona, Verona, Italy; b Institute for Social and Preventive Medicine, University of Bern, Bern, Switzerland; c Istituto di Ricerche Farmacologiche Mario Negri IRCCS, Milano, Italy; d Department of Neuroscience, Rehabilitation, Ophthalmology, Genetics, Maternal and Child Health (DiNOGMI), Section of Psychiatry, University of Genoa, Genoa, Italy; e IRCCS Ospedale Policlinico San Martino, Genoa, Italy; f Department of Clinical and Experimental Medicine, Institute of Psychiatry, University of Catania, Catania, Italy; g Department of Medicine and Surgery, University of Milano-Bicocca, Monza, Italy; h Department of Medicine and Surgery, Section of Psychiatry, University of Insubria, Varese, Italy; i Psychiatry Unit, Department of Health Sciences, University Magna Graecia of Catanzaro, Catanzaro, Italy; j Psychiatry Unit, Department of Medical and Surgical Sciences, University Magna Graecia of Catanzaro, Catanzaro, Italy; k Outpatient Clinic for Clinical Research and Treatment of Eating Disorders, University Hospital Renato Dulbecco, Catanzaro, Italy; l Department of Neuroscience and Rehabilitation, Institute of Psychiatry, University of Ferrara, Ferrara, Italy; m University Hospital Psychiatry Unit, Integrated Department of Mental Health and Addictive Behavior, University S. Anna Hospital and Health Trust, Ferrara, Italy; n Department of Mental Health and Addiction, ASST Santi Paolo e Carlo, Milan, Italy; o Department of Health Sciences, Università degli Studi di Milano, Milan, Italy; p Department of Neurosciences, Imaging and Clinical Sciences, University “G. D'Annunzio”, Chieti, Italy; q Santa Chiara Hospital, Verona, Italy; r Department of Life, Health and Environmental Sciences, University of L'Aquila, L'Aquila, Italy; s Pharmacy Unit, Azienda Ospedaliera Universitaria Integrata di Verona, Verona, Italy; t Department of Human Neurosciences, Sapienza University of Rome, Rome, Italy

**Keywords:** Older adults, Major depressive disorder, Vortioxetine, Serotonin selective reuptake inhibitors, Tolerability, Adverse events

## Abstract

**Background:**

Major depressive disorder (MDD) is prevalent and disabling among older adults. Standing on its tolerability profile, vortioxetine might be a promising alternative to selective serotonin reuptake inhibitors (SSRIs) in such a vulnerable population.

**Methods:**

We conducted a randomised, assessor- and statistician-blinded, superiority trial including older adults with MDD. The study was conducted between 02/02/2019 and 02/22/2023 in 11 Italian Psychiatric Services. Participants were randomised to vortioxetine or one of the SSRIs, selected according to common practice. Treatment discontinuation due to adverse events after six months was the primary outcome, for which we aimed to detect a 12% difference in favour of vortioxetine. The study was registered in the online repository clinicaltrials.gov (NCT03779789).

**Findings:**

The intention-to-treat population included 179 individuals randomised to vortioxetine and 178 to SSRIs. Mean age was 73.7 years (standard deviation 6.1), and 264 participants (69%) were female. Of those on vortioxetine, 78 (44%) discontinued the treatment due to adverse events at six months, compared to 59 (33%) of those on SSRIs (odds ratio 1.56; 95% confidence interval 1.01–2.39). Adjusted and per-protocol analyses confirmed point estimates in favour of SSRIs, but without a significant difference. With the exception of the unadjusted survival analysis showing SSRIs to outperform vortioxetine, secondary outcomes provided results consistent with a lack of substantial safety and tolerability differences between the two arms. Overall, no significant differences emerged in terms of response rates, depressive symptoms and quality of life, while SSRIs outperformed vortioxetine in terms of cognitive performance.

**Interpretation:**

As opposed to what was previously hypothesised, vortioxetine did not show a better tolerability profile compared to SSRIs in older adults with MDD in this study. Additionally, hypothetical advantages of vortioxetine on depression-related cognitive symptoms might be questioned. The study's statistical power and highly pragmatic design allow for generalisability to real-world practice.

**Funding:**

The study was funded by the 10.13039/501100003197Italian Medicines Agency within the “2016 Call for Independent Drug Research”.

**Research in context**

**Evidence before this study**

After searching PubMed for randomised and meta-analytical evidence on vortioxetine in older adults (syntax: ((older adults [Title/Abstract] OR elder∗[Title/Abstract]) AND (vortioxetine [Title/Abstract])); Filters: Meta-Analysis, Randomised Controlled Trial; from inception to 14/09/2023), we found one network meta-analysis on older adults with depression, showing no tolerability differences between vortioxetine and selective serotonin reuptake inhibitors based on indirect evidence (certainty of evidence not available for tolerability), as only one randomised trial comparing vortioxetine, duloxetine and placebo in 452 individuals was included. Further, one small trial compared vortioxetine and sertraline in 60 individuals, showing no significant tolerability differences.

**Added value of this study**

To our knowledge, this is the largest randomised trial specifically comparing vortioxetine and selective serotonin reuptake inhibitors in older adults with major depression under real-world clinical conditions, according to a highly pragmatic design.

**Implications of all the available evidence**

Results from the VESPA study disconfirms the notion of vortioxetine being more tolerable than selective serotonin reuptake inhibitors in older adults, which we believe requires update of evidence-based guidelines. Still, future large observational studies might inform on rare and potentially serious adverse events of fragile populations taking antidepressants.

## Introduction

Major depressive disorder (MDD) is one of the world's most disabling conditions,^1^ affecting approximately 4% of older adults in the community^2^ and up to half of those admitted to nursing homes and hospitals.^3^^,^^4^ In older adults, MDD is associated with several undesirable outcomes, including reduced adherence to treatment, poorer medical outcomes,^5^ increased risk of cognitive impairment and dementia,^6^ and reduced life expectancy for several reasons, including increased risk of suicide.^7^ Selective serotonin reuptake inhibitors (SSRIs) are generally considered effective and safe in both adults^8^ and older adults^9^ with MDD, and most guidelines recommend them as first-line pharmacological treatment for older adults.^10^^,^^11^ However, older adults may be particularly vulnerable to adverse events due to ageing itself, medical comorbidities, multiple treatments, and a high risk of pharmacological interactions.^12^^,^^13^ Additionally, several adverse events associated with SSRIs, such as hyponatraemia, postural hypotension, falls, gastrointestinal bleeding, and sexual dysfunction, are relatively common in this population.^14^^,^^15^ Alternatives to SSRIs for older adults are lacking, as safety and tolerability concerns are even greater for tricyclic antidepressants, serotonin and norepinephrine reuptake inhibitors, mirtazapine and bupropion.^15^

Vortioxetine was approved by the Food Drug Administration (FDA) and European Medicines Agency (EMA) for the treatment of MDD in 2013.^16^^,^^17^ Its pharmacological profile includes antagonism for hydroxytryptamine (HT) receptors 5-HT3, 5-HT1D and 5-HT7, partial agonism for 5-HT1B, and agonism for 5-HT1A.^18^ Unlike SSRIs, its mechanism of action includes both direct modulation of serotoninergic receptors and inhibition of the serotonin transporter, and is therefore classified in the World Health Organization (WHO) ATC/DDD Index 2018 in the group “other antidepressants”.^19^ According to the available data on its pharmacokinetic and pharmacodynamic mechanisms, vortioxetine may have a more favourable tolerability profile.^16^^,^^20^^,^^21^ Moreover, possible beneficial effects on depression-related cognitive dysfunction have been highlighted.^22^^,^^23^ These properties may make vortioxetine suitable for vulnerable populations, including those with medical comorbidities and older adults.^21^

A network meta-analysis in older adults with MDD^24^ showed no differences between vortioxetine and other SSRIs in terms of efficacy, dropouts due to adverse events, and individual adverse events, although results were solely based on indirect estimates, as only one randomised controlled trial (RCT) on vortioxetine vs. placebo and duloxetine (452 individuals) was included.^25^ More recently, a small RCT comparing vortioxetine and sertraline in 60 older adults with MDD found no differences in efficacy and tolerability, although the small sample size significantly limits statistical power and the overall confidence in the estimates.^26^ A pairwise meta-analysis pooling data on the subgroup of adults over 55 years of age from available RCTs in the adult population^27^ showed no significant tolerability differences of vortioxetine compared to placebo. Such data on older adults are consistent with those from the adult population, where a large network meta-analysis^8^ showed similar efficacy and tolerability for vortioxetine and SSRIs, but also in this case direct comparisons between these treatments were not available. More recently, two RCTs, primarily aimed to assess cognitive performance in adults with MDD,^28^^,^^29^ showed no differences between vortioxetine and SSRIs (paroxetine and escitalopram, respectively) in terms of cognitive performance, efficacy, and tolerability.

Therefore, the available evidence does not provide a clear indication of whether vortioxetine might have tolerability advantages over SSRIs in older adults, as might be hypothesised from its pharmacological properties. Despite this lack of empirical data, vortioxetine is one of the most commonly recommended antidepressants for people with comorbid conditions,^30^ and is one of the safest choices for older adults according to the Maudsley Prescribing Guidelines.^31^ On these grounds, we conducted a RCT with the primary aim of assessing the superiority of vortioxetine compared to SSRIs in terms of tolerability (i.e., treatment discontinuation due to adverse events) in older adults with MDD under real-world clinical conditions.

## Methods

### Study design, ethics and participants

The VESPA (Vortioxetine in the Elderly vs. SSRIs: A Pragmatic Assessment) study is a pragmatic, open-label, randomised, parallel-group, multicentre, superiority trial. The study was conducted between February 2nd, 2019 and February 22nd, 2023 in 11 Italian Psychiatric Services, namely: University of Verona, University of Catania, Magna Graecia University (Catanzaro), University of Ferrara, University of Genoa, University of Chieti-Pescara, University of Insubria (Varese), University of L'Aquila, University La Sapienza (Rome), University of Milan, University of Milano-Bicocca. The study protocol (Supplement A) was designed according to the principles of the CONSORT statement (extended version for pragmatic trials),^32^ the SPIRIT 2013 statement,^33^ and globally accepted standards defined by the ICH E6 Guideline for Good Clinical Practice (1 May 1996) and the Declaration of Helsinki.^34^ Participants' data were managed and safeguarded in accordance with the European Data Protection Regulation 2016/679.^35^ The participant information sheet and the informed consent form are available as additional online material (Supplement C and D, respectively). The study was firstly approved for the coordinating centre (University of Verona) by the Ethics Committee for Clinical Research of Verona and Rovigo (protocol 61211 of the 19/09/2018; protocol version 1.5 of the 09/06/2018) (Supplement D), and thereafter by the local Ethics Committee of each recruiting centre. The coordinating centre monitored the trial according to the Good Clinical Practice and International Conference on Harmonisation guidelines.^36^ The study was registered in the online repository clinicaltrials.gov (NCT03779789) and the study protocol was published in advance.^37^ Deviations from the protocol are reported in the Supplement E. The VESPA study was designed according to principles of pragmatism, in order to resemble real-world populations, practices and interventions as much as possible, as shown by high scores in most domains of the PRagmatic Explanatory Continuum Indicator Summary (PRECIS-2) (Supplement F).^37^

Eligible participants were aged 65 years or older; were willing to participate by signing an informed consent form; and suffered from an episode of MDD at the time of recruitment, based on clinical judgment guided by the Diagnostic and Statistical Manual for Mental Disorders–fifth edition (DSM-5) criteria. Participants with a formal diagnosis of dementia (any type and stage), schizophrenia, or bipolar disorder were excluded. Participants were included if treatment with an antidepressant was considered clinically appropriate, and there was uncertainty about which trial treatment would be best for the participant. Individuals with clinical conditions or treatments contraindicating the use of oral vortioxetine or SSRIs, according to clinical judgment, were excluded. Otherwise, concomitant medical and/or psychotropic medications at the time of randomisation were allowed, except for agents with antidepressant properties, namely antidepressants, second generation antipsychotics, and lithium. We chose the class of SSRIs as the comparator, rather than a single SSRI, because these agents have similar efficacy and tolerability profiles in both the general population and older adults,^8^^,^^38^ and this allowed for broader eligibility criteria and flexibility in the intervention, in line with the principles of pragmatism in clinical trials.^39^

### Randomisation and masking

Participants were randomly allocated to vortioxetine or to SSRIs with an allocation ratio of 1:1. We employed the web-based application RedCap©,^40^ which allowed for a centralised procedure based on an allocation sequence of treatments randomly permuted in blocks of constant size (random even sizes between 2 and 8). This sequence and the block size were concealed to study investigators. Allocation was stratified by recruiting centre. By using RedCap©, investigators were able to screen participants for inclusion, administer questionnaires and rating scales maintaining the blindness to treatment allocation, and randomise them. After randomisation and throughout the trial, patients and clinicians were aware of treatment allocation, while outcome assessors and biostatisticians performing the analyses operated under blind conditions.

### Procedures

Recruiting psychiatrists consecutively enrolled participants from in- and outpatient clinics of each recruiting centre. The first visit took place according to routine clinical practice. Within the same visit, potentially eligible individuals were thoroughly informed on the nature of the study and its procedures, and then asked to provide a signed consent to participate. These individuals underwent baseline assessment, which included socio-demographic, clinical and anamnestic data, as well as rating scales. The application RedCap© allowed randomisation only after baseline data were collected, to preserve allocation concealment. Participants were therefore randomised to either vortioxetine or one SSRI. Recruiting psychiatrists selected the SSRIs which they considered more appropriate according to participants' individual characteristics, choosing among those marketed in Italy (i.e., sertraline, citalopram, escitalopram, paroxetine, fluoxetine, fluvoxamine). A flexible dosing schedule, within the licensed dose range and in line with the Summary of Product Characteristics registered in the databank of Italian Medicines Agency *(Agenzia Italiana del Farmaco—AIFA)* was allowed (Supplement A, page 8). The choice of formulation (i.e., tablets vs. drops) was made by clinicians based on clinical and practical considerations, including participants’ preferences. Aiming to resemble clinical practice as much as possible, no extra measures aimed at optimising treatment adherence were implemented. We collected clinical data and rating scales at baseline and at each follow-up visit, which took place at one, three, and six months after randomisation. Clinical data included current and previous medical and psychiatric comorbidities and treatments. Validated rating scales included the Montgomery–Åsberg Depression Rating Scale (MADRS)^41^ to assess depressive symptoms, the EuroQol Group 5-dimension (EQ-5D)^42^ to assess health-related quality of life, the Short Blessed Test (SBT)^43^ to assess cognitive performance, and the Charlson Age-Comorbidity Index (CACI)^44^ to assess the presence and severity of medical comorbidities. Additionally, the Antidepressant Side-Effect Checklist (ASEC)^45^ was administered only at follow-up visits.

Data collection and digital storage thorough RedCap©, as well as statistical analyses, were managed by the Italian not-for-profit biomedical research organisation *Istituto di Ricerche Farmacologiche Mario Negri IRCCS*. RedCap© allowed for an immediate data validation at the moment of data entering. Moreover, a set of electronic and manual quality checks were performed.

### Outcomes

The primary outcome was the number of participants discontinuing the assigned antidepressant for more than two consecutive weeks following the occurrence of any adverse event over the 6-months follow-up. Treatment discontinuation due to adverse events was considered a pragmatic proxy of tolerability,^46^ as it occurs when adverse events reach an unbearable burden as perceived by patients, caregivers, or clinicians. Secondary outcomes included: (1) number of participants discontinuing from allocated treatment due to any cause; (2) overall mortality; (3) suicidal behaviours (including episodes of non-suicidal self-injury, suicide attempt, and death by suicide); (4) adverse events, measured through the ASEC mean score at each time point and the mean of the highest scores obtained throughout the study by each participant; (5) response to treatment, defined as a reduction of at least 50% of the baseline score of the MADRS; (6) efficacy, measured as MADRS mean scores at each time point; (7) quality of life, measured as EQ-5D mean scores at each time point; (8) cognitive performance, measured as SBT mean scores at each time point. In terms of safety assessment, in accordance to the EU regulation about pharmacovigilance in clinical research^47^ and the EC Directive 2001/20/EC,^48^ an *ad hoc* Serious Adverse Events (SAE) form was employed and forwarded to the coordinating centre (University of Verona) as soon as the adverse event occurred.

### Statistical analysis

For the power analysis, we used a two-sided Z test with pooled variance, and a significance level targeted at 5%, according to the methodology described by Pocock.^49^ Based on available literature in older adults with MDD, we expected the vortioxetine group to show a clinically significant advantage over SSRIs by reducing the rate of antidepressant discontinuation due to adverse events from about 17%^9^ to about 5%.^25^^,^^50^^,^^51^ Therefore, we calculated a sample size of 276 participants (138 in each group) to achieve 90% power to detect a difference of 12% between the two discontinuation proportions in favour of vortioxetine. Further, we assumed that about 23% of participants would fail to provide valid data for the primary outcome within 6 months, based on dropout rates from available studies on vortioxetine in the older adults.^51^ Therefore, we set our target sample size at 358 participants (179 in each group) to enrol in order to obtain at least 276 evaluable individuals.

For the analysis of both primary and secondary outcomes we used the intention-to-treat (ITT) population, which consisted of all randomised participants, with the only exception of those withdrawing study consent. Following a conservative approach, we assumed that individuals with missing data for the primary outcome had discontinued due to adverse events. All analyses were performed using Statistical Analysis System (SAS) version 9.1.4. Nominal value for statistical significance was set at 0.05, two-tailed. As the study was not powered to test secondary outcomes, the results of these tests should be interpreted with caution and considered exploratory only.

For the analysis of binary outcomes, we compared the two arms using a Generalized Linear Mixed Model (GLMM) with centre as a random effect. For confirmatory purposes only, the primary outcome was also analysed using a per-protocol (PP) approach, which was restricted to individuals with primary outcome assessment available at the end of follow-up, and who did discontinue the antidepressants for reasons not related to the primary outcome. Additionally, we performed a multivariable analysis through GLMM with centre as a random effect and adjusting for the potential confounding effect of prognostic factors: sex, age, housing conditions, marital status, working conditions, years of education, MADRS baseline score, time of titration, and ratio between prescribed daily dose and defined daily dose (PDD/DDD), according to the WHO ATC/DDD classification (https://www.whocc.no/atc_ddd_index/). For continuous outcomes, we compared the two arms using GLMM with centre as a random effect and the baseline score as a covariate. Additionally, a Cox proportional hazard model was used to explore time to treatment discontinuation due to adverse events according to a “worst-case scenario”, where missing data for the primary outcome were assumed to be discontinuations due to adverse events (as for the analysis of the primary outcome), and a “best-case scenario”, where participants with missing data for the primary outcome were considered as continuing treatment. The proportional hazard assumption of the effects was tested. As an additional analysis, the Wilcoxon Rank–Sum Test for two samples (or Mann–Whitney test) was used to compared the mean change score from baseline to 6 months.

Adverse events were tabulated. For continuous outcomes, missing rating scales scores were imputed using both the Last Observation Carried forward (LOCF) and Rubin's approach to multiple imputation.^52^ First, by using the SAS’ Procedure Multiple Imputation (PROC MI), missing data were filled in n times using chained equations to generate n complete data sets (we created n = 10 complete data sets), with predictive mean matching method for numeric variables, and discriminant function method for categorical variables. Variables used for the imputations were: age, sex, education, centre, living condition, retirement status, presence of other psychiatric comorbidities, drug dosage and titration, and scores at MADRS, CACI, ASEC, EQ-5D, and SBT. Then the 10 complete data sets were analysed using the GLMM approach. Finally, the results from the 10 complete data sets were combined via the SAS’ Myanalyze procedure to obtain a single inferential result for each outcome. The 6-month estimate was compared between the two arms with an analysis of covariance with baseline value as an additional covariate, or with Mann–Whitney test on changes, according to the variable's distribution. For the analysis of the SBT score, we conducted a post-hoc analysis removing participants who were diagnosed with dementia throughout follow-up.

### Role of the funding source

The VESPA study was funded by the Italian Medicines Agency (*Agenzia Italiana del Farmaco—AIFA*) within the 2016 call for Independent Research on Drugs (code: 2016-0234923). The funder had no role in the study design; patient recruitment; data collection, analysis, and interpretation; writing the study report; and in the decision to submit the paper for publication. All authors had full access to all the data in the study and accept responsibility for the decision to submit for publication.

## Results

**Fig. 1:**
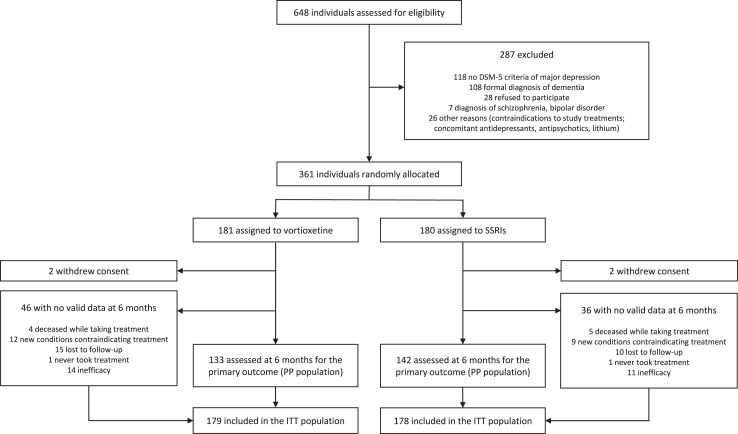
Study flow-chart.

Participants were recruited between February 12, 2019 and August 22, 2022, and the final study visit took place on February 27, 2023. Fig. 1 shows the study flow-chart. A total of 648 individuals were assessed for eligibility. Of these, 259 did not meet eligibility criteria. Of those eligible, 28 did not agree to take part to the trial, and 361 agreed to participate by signing the written informed consent and underwent randomisation (Fig. 1). Two participants for each arm withdrew study consent during follow-up and were excluded from the analyses. Therefore, the ITT population was composed of 357 participants. The mean age was 73.7 (standard deviation (SD) 6.1); 246 participants (69%) were female; 201 (56%) suffered from one or more medical conditions, and 65 (18%) suffered from a psychiatric comorbidity in addition to the diagnosis of MDD, including mostly anxiety disorders. Table 1 shows the main socio-demographic and clinical characteristics of participants of the two treatment groups, which appeared to be well-balanced. The most prescribed SSRIs was sertraline (106 participants, 60%). By the end of the study, 275 participants (133 on vortioxetine and 142 on SSRIs) provided valid data on the primary outcome (Fig. 1), consistently with what was expected according to the study protocol (i.e., 276 participants). These participants are indicated as the “PP population”. For the remaining 82 participants, valid data on the primary outcome at 6 months were not available for various reasons, including: never taking study treatment (1 vortioxetine, 1 SSRIs); deceased while taking study treatment (4 vortioxetine, 5 SSRIs); new conditions contraindicating study treatment (12 vortioxetine, 9 SSRIs); treatment discontinuation due to inefficacy (14 vortioxetine, 11 SSRIs); lost to follow-up (15 vortioxetine, 10 SSRIs). Conservatively, these participants were included in the ITT analysis assuming that the event “discontinuation due to adverse events” (primary outcome) had occurred. Throughout the study, several participants received additional treatments, including other antidepressants (N = 24, 7%), antipsychotics (N = 27, 8%), mood stabilisers (N = 28, 8%), benzodiazepines (N = 132, 37%), and psychological support (N = 10, 3%), with no imbalances between treatment arms (Supplement G).

**Table 1:** Baseline characteristics of study participants (intention-to-treat population).

Variable	Vortioxetine (N = 179)	SSRI (N = 178)
Females, n (%)	118 (65.9)	128 (71.9)
Age, mean (SD)	73.8 (6.3)	73.7 (5.9)
Living conditions, n (%)		
Alone	51 (28.5)	60 (34.1)
Alone with domestic assistance	4 (2.2)	3 (1.7)
With family	124 (69.3)	111 (63.1)
Nursing home	–	2 (1.1)
*Missing*	–	*2*
Married, n (%)	97 (55.4)	97 (55.7)
*Missing*	*4*	*4*
Employment, n (%)		
Employed	10 (5.6)	11 (6.3)
Unemployed	13 (7.3)	11 (6.3)
Retired	155 (87.1)	153 (87.4)
*Missing*	*1*	*3*
Years of education, mean (SD)	9.3 (4.0)	9.3 (4.3)
*Missing*	*5*	*8*
Setting of recruitment, n (%)		
Psychiatry outpatient clinic	161 (89.9)	149 (83.7)
Psychiatry ward	7 (3.9)	16 (9.0)
Medical/surgical ward	11 (6.1)	13 (7.3)
Medical comorbidities, n (%)		
None	77 (43.0)	78 (43.8)
One medical comorbidity	48 (26.8)	57 (32.0)
Two or more medical comorbidities	54 (30.2)	43 (24.2)
Type of medical comorbidity^a^, n (%)		
Diabetes	33 (18.4)	27 (15.2)
Liver disease	9 (5.0)	13 (7.3)
Peripheral vasculopathy	42 (23.5)	35 (19.7)
Cerebrovascular events	11 (6.1)	10 (5.6)
Myocardial infarction	13 (7.3)	10 (5.6)
Congestive heart failure	13 (7.3)	9 (5.1)
Chronic obstructive pulmonary disease	15 (8.4)	13 (7.3)
Connective tissue disease	20 (11.2)	22 (12.4)
Cancer	21 (11.7)	15 (8.4)
CACI index score, mean (SD)	4.3 (1.8)	4.1 (1.9)
*Missing*	*1*	–
SBT score, mean (SD)	4.4 (5.1)	4.6 (5.2)
*Missing*	–	*1*
At least one psychiatric comorbidity, n (%)	39 (21.8)	26 (14.6)
Type of psychiatric comorbidity, n (%)		
Anxiety disorders	30 (16.7)	19 (10.7)
Adjustment disorder	2 (1.1)	2 (1.1)
Disorders due to use of alcohol	4 (2.2)	4 (2.2)
Personality disorders	1 (0.6)	2 (1.1)
Eating disorders	–	1 (0.6)
Somatic symptoms disorders	1 (0.6)	2 (1.1)
Sleep disorders	2 (1.1)	–
Years from onset of psychiatric illness, mean (SD)	10.4 (13.9)	10.3 (15.1)
*Missing*	*11*	*12*
MADRS baseline score, mean (SD)	27.3 (8.1)	27.5 (8.2)
Inner tension (MADRS item #3), n (%)	79 (44.1)	79 (44.4)
Suicidality (current), n (%)	8 (4.5)	9 (5.1)
EQ-5D, mean (SD)	49.2 (19.1)	45.6 (18.6)
*Missing*	*1*	–
Type of SSRI prescribed, n (%)		
Sertraline	–	106 (59.6)
Escitalopram	–	27 (15.2)
Paroxetine	–	22 (12.4)
Citalopram	–	18 (10.1)
Fluoxetine	–	4 (2.2)
Fluvoxamine	–	1 (0.6)

Legend: CACI, Charlson Age Comorbidity Index; EQ-5D, EuroQol Group 5-dimension health-related quality of life; IQR, Inter-Quartile Range; N, number of participants randomised: MADRS, Montgomery–Åsberg Depression Rating Scale; SD, standard deviation; SBT, Short Blessed Test; SSRI, selective serotonin reuptake inhibitor.

a Only medical comorbidities occurring in at least 5% of the recruited participants were reported.

**Fig. 2:**
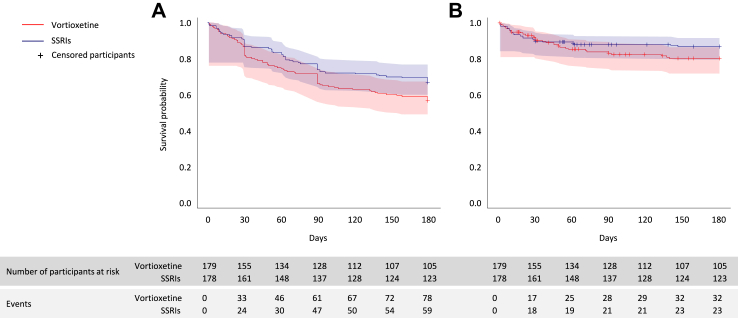
Time to discontinuation due to adverse events. A) Shows the worst-case scenario, where missing data for the primary outcome were assumed to be discontinuations due to adverse events. B) Shows the best-case scenario, where missing data for the primary outcome were assumed to be participants continuing treatment. Legend: SSRI, selective serotonin reuptake inhibitor.

According to the ITT analysis, 78 out of 179 participants (44%) discontinued the treatment due to adverse events at 6 months in the vortioxetine arm, compared to 59 out of 178 (33%) participants in the SSRI arm, with evidence of a statistically significant difference in favour of the latter (N = 357; odds ratio (OR) 1.56; 95% CI 1.01–2.39; p = 0.042) (Table 2). Secondary analyses performed on this outcome, including the ITT analyses at 1 and 3 months, the PP analysis at 6 months, and the mixed models on both ITT and PP populations adjusting for multiple covariates, confirmed a point estimate in favour of the SSRIs, but none of them showed a significant difference between the two arms (Table 2). The Kaplan–Meier survival curve of the “worst-case scenario” showed that time to discontinuation due to adverse events significantly differed between the two arms (N = 357; hazard ratio (HR) 1.42; 95% CI 1.01–1.99; p = 0.045), however, this finding was not confirmed in the adjusted analysis or in the “best-case scenario”, where participants with missing data for the primary outcome were considered as continuing treatment (Fig. 2 and Table 2). The most common adverse events causing treatment discontinuation were vomiting (6 individuals in each arm), anxiety/irritability (6 vortioxetine, 3 SSRIs), confusion (2 vortioxetine, 3 SSRIs), and diarrhoea (3 vortioxetine, 0 SSRIs) (Table 3). When considering adverse events of any severity, nausea was the most common, although not associated with treatment discontinuation (Table 3). The decision of discontinuing the treatment due to adverse events was most commonly taken by study participants without consulting the study psychiatrist (14/32 (43.8%) on vortioxetine vs. 10/23 (43.5%) on SSRIs), or by other doctors not involved in the study (i.e., general practitioners or other specialists consulted by patients) (6/32 (18.8%) on vortioxetine vs. 4/23 (17.4%) on SSRIs).In a minority of cases this decision was taken by the study psychiatrist (2/32 (6.3%) on vortioxetine vs. 3/23 (13.0%) on SSRIs), while for several cases this information was missing (10/32 (31.3%) on vortioxetine vs. 6/23 (26.1%) on SSRIs).

**Table 2:** Study outcomes.

Binary outcomes	Vortioxetine	SSRIs	Unadjusted			Adjusted		
	n/N (%)	n/N (%)	k	OR (95% CI)	p	k	OR (95% CI)	p
**Discontinuation due to AEs**								
1 month	37/179 (20.7%)	28/178 (15.7%)	357	1.40 (0.81–2.41)	0.23	337	1.01 (0.54–1.86)	0.99
3 months	66/179 (36.9%)	52/178 (29.2%)	357	1.42 (0.91–2.23)	0.13	337	1.25 (0.75–2.06)	0.39
6 months (PRIMARY OUTCOME)	78/179 (43.6%)	59/178 (33.1%)	357	1.57 (1.01–2.44)	*0.042*	337	1.25 (0.76–2.06)	0.38
6 months (per protocol)	32/133 (24.1%)	23/142 (16.2%)	275	1.67 (0.91–3.08)	0.097	262	1.02 (0.50–2.09)	0.96
**Time to discontinuation due to AEs**^a^								
Survival analysis (worst-case scenario) [HR]	–	–	357	1.42 (1.01–1.99)	*0.045*	337	1.11 (0.76–1.61)	0.061
Survival analysis (best-case scenario) [HR]	–	–	357	1.44 (0.84–2.46)	0.18	337	0.95 (0.52–1.72)	0.85
**Discontinuation due to any cause**^b^								
1 month	34/179 (19.0%)	27/178 (15.2%)	357	1.31 (0.75–2.29)	0.34	337	0.97 (0.52–1.81)	0.93
3 months	61/179 (34.1%)	50/178 (28.1%)	357	1.33 (0.84–2.09)	0.22	337	1.23 (0.74–2.04)	0.43
6 months	74/179 (41.3%)	58/178 (32.6%)	357	1.46 (0.94–2.27)	0.087	337	1.27 (0.78–2.07)	0.34
**Mortality**								
Overall mortality	5/179 (2.8%)	5/178 (2.8%)	–	–	–	–	–	–
**Suicidal behaviours**								
Non-suicidal self-injury	0/179 (0.0%)	0/178 (0.0%)	–	–	–	–	–	–
Suicide attempt	1/179 (0.5%)	0/178 (0.0%)	–	–	–	–	–	–
Death by suicide	0/179 (0.0%)	1/178 (0.5%)	–	–	–	–	–	–
**Serious adverse events**								
6 months	11/179 (6.1%)	7/178 (3.9%)	–	–	–	–	–	–
**Responders**								
1 month	57/179 (31.8%)	64/178 (36.0%)	357	0.82 (0.53–1.30)	0.42	337	0.82 (0.51–1.33)	0.42
3 months (LOCF)	84/179 (46.9%)	100/178 (56.2%)	357	0.69 (0.45–1.05)	0.084	337	0.62 (0.39–1.00)	*0.047*
6 months (LOCF)	94/179 (52.5%)	107/178 (60.1%)	357	0.73 (0.48–1.12)	0.15	337	0.64 (0.40–1.02)	0.061
**Continuous outcomes**	**mean (SD)**	**mean (SD)**	**k**	**Estimate (95% CI)**	**p**	**k**	**Estimate (95% CI)**	**p**

**ASEC**								
Highest score	7.60 (5.23)	7.95 (5.18)	331	−0.48 (−1.51 to 0.55)	0.36	313	−0.31 (−1.45 to 0.83)	0.59
Score 1 month	5.64 (4.88)	6.17 (5.04)	327	−0.63 (−1.62 to 0.37)	0.22	309	−0.51 (−1.61 to 0.59)	0.36
Score 3 months (LOCF)	5.44 (4.62)	5.35 (5.02)	331	−0.03 (−0.98 to 0.93)	0.95	313	0.04 (−1.01 to 1.09)	0.94
Score 6 months (LOCF)	4.95 (4.67)	5.37 (4.52)	331	−0.54 (−1.45 to 0.37)	0.25	313	−0.45 (−1.42 to 0.53)	0.37
Score 6 months (multiple imputation)	–	–	357	−0.54 (−1.58 to 0.50)	0.31	357	−0.63 (−1.71 to 0.46)	0.25
**MADRS**								
Baseline	27.3 (8.14)	27.5 (8.21)	–	–	–		–	–
Score 1 month (LOCF)	18.7 (10.33)	18.1 (10.55)	357	0.69 (−1.22 to 2.60)	0.48	337	1.08 (−1.01 to 3.16)	0.31
Score 3 months (LOCF)	15.7 (10.18)	13.9 (9.77)	357	1.76 (−0.15 to 3.67)	0.071	337	2.14 (0.09–4.18)	*0.041*
Score 6 months (LOCF)	14.6 (10.39)	13.0 (9.48)	357	1.68 (−0.26 to 3.61)	0.089	337	2.21 (0.14–4.29)	*0.037*
Score 6 months (multiple imputation)	–	–	357	0.79 (−1.04 to 2.62)	0.40	357	1.66 (−0.16 to 3.49)	0.074
**EQ-5D**								
Baseline	49.2 (19.15)	45.6 (18.62)	–	–	–		–	–
Score 1 month (LOCF)	55.1 (20.42)	56.9 (19.60)	356	−2.87 (−6.82 to 1.07)	0.15	336	−3.43 (−7.68 to 0.82)	0.11
Score 3 months (LOCF)	58.9 (19.95)	60.5 (19.33)	356	−2.40 (−6.35 to 1.54)	0.23	336	−2.68 (−6.94 to 1.58)	0.22
Score 6 months (LOCF)	59.2 (20.90)	62.2 (19.18)	356	−3.49 (−7.59 to 0.61)	0.095	336	−3.72 (−8.10 to 0.65)	0.095
Score 6 months (multiple imputation)	–	–	357	−2.91 (−7.42 to 1.60)	0.20	357	−3.18 (−7.79 to 1.42)	0.17
**SBT**								
Baseline	4.41 (5.05)	4.63 (5.16)	–	–	–		–	–
Score 1 month (LOCF)	4.37 (5.31)	4.80 (5.81)	356	−0.27 (−1.14 to 0.60)	0.54	337	−0.04 (−0.98 to 0.89)	0.93
Score 3 months (LOCF)	4.10 (5.13)	3.93 (5.21)	356	0.30 (−0.54 to 1.14)	0.49	337	0.19 (−0.71 to 1.09)	0.67
Score 6 months (LOCF)	4.55 (6.09)	3.55 (5.10)	356	1.14 (0.21–2.06)	*0.016*	337	1.24 (0.26–2.22)	*0.014*
Score 6 months (multiple imputation)	–	–	357	1.01 (−0.03 to 2.05)	0.057	357	0.92 (−0.17 to 2.02)	0.098

Analyses were conducted on the intention-to-treat (ITT) population unless otherwise specified.

For negative binary outcomes, OR < 1 favours vortioxetine, for positive binary outcomes, OR > 1 favours vortioxetine. For continuous outcomes, estimates >0 for EQ-5D, and <0 for ASEC, MADRS and SBT favour vortioxetine. p-values below 0.5 are reported in italics.

Unadjusted analyses: Generalized Linear Mixed Model (GLMM) model with random effect for recruiting centre.

Adjusted analyses: Generalized Linear Mixed Model (GLMM) model with random effect for recruiting centre and the following covariates: sex (male vs. female), age (continuous), housing conditions (with family vs. others), marital status (married vs. not married), working condition (retired from work vs. others), years of education (up to 8 years vs. 9 or more years), CACI score (continuous), psychiatric comorbidities (none vs. one or more), baseline MADRS score (continuous), time of titration in days (continuous), ratio between prescribed daily dose and defined daily dose (PDD/DDD, continuous). For continuous outcomes of MADRS, EQ-5D and SBT, the GLMM models include also the baseline score.

Legend: AE, adverse event; ASEC, Antidepressant Side Effect Checklist; CI, confidence interval; HR, hazard ratio; EQ-5D, EuroQol Group 5-dimension health-related quality of life; k, overall number of participants included in the analysis; MADRS, Montgomery–Åsberg Depression Rating Scale; n, number of events; N, number of individuals in the analysis; NE, not estimable; OR, odds ratio; SD, standard deviation; SSRI, selective serotonin reuptake inhibitor.

a In the “worst-case scenario” missing data for the primary outcome were assumed to be discontinuations due to adverse events, while in the “best-case scenario” missing data for the primary outcome were assumed to be participants continuing treatment.

b In this analysis, the number of participants discontinuing due to any cause is lower than those discontinuing due to adverse events. This is because the analysis of discontinuations due to any cause includes only those discontinuations due to adverse events that are included in the per-protocol analysis of the primary outcome, while the analysis of discontinuations due to adverse events conservatively includes all discontinuations.

**Table 3:** Adverse events occurring in 2% or more of participants (in at least one study arm), and/or associated with treatment discontinuation.

Adverse events	Any		Causing treatment discontinuation (primary outcome)	
	Vortioxetine (N = 179)	SSRIs (N = 178)	Vortioxetine (N = 179)	SSRIs (N = 178)
Gastrointestinal and nutritional				
Nausea	15 (8.4%)	14 (7.9%)	–	–
Vomiting	9 (5.0%)	6 (3.4%)	6 (3.3%)	6 (3.4%)
Diarrhoea	6 (3.3%)	1 (0.5%)	3 (1.7%)	–
Constipation	2 (1.1%)	3 (1.7%)	1 (0.5%)	1 (0.5%)
Oral dysesthesia	3 (1.7%)	–	1 (0.5%)	–
Epigastric pain	3 (1.7%)	5 (2.8%)	–	1 (0.5%)
Loss of appetite	4 (2.2%)	–	–	–
Weight loss	1 (0.5%)	–	1 (0.5%)	–
Psychiatric				
Psychomotor agitation	4 (2.2%)	–	–	–
Anxiety, irritability	13 (7.3%)	5 (2.8%)	6 (3.3%)	3 (1.7%)
Confusion	3 (1.7%)	5 (2.8%)	2 (1.1%)	3 (1.7%)
Abnormal dreams	1 (0.5%)	–	1 (0.5%)	–
Insomnia	2 (1.1%)	6 (3.4%)	–	–
Neurological				
Somnolence, daytime sedation	1 (0.5%)	9 (5.0%)	1 (0.5%)	2 (1.1%)
Vertigo, dizziness	5 (2.8%)	7 (3.9%)	1 (0.5%)	2 (1.1%)
Headache	7 (3.9%)	5 (2.8%)	–	2 (1.1%)
Paraesthesia	1 (0.5%)	–	1 (0.5%)	–
Presyncope	1 (0.5%)	–	1 (0.5%)	–
Fatigue	2 (1.1%)	11 (6.2%)	–	2 (1.1%)
Tremor	3 (1.7%)	4 (2.2%)	–	–
Cardiovascular				
Hypertension	4 (2.2%)	2 (1.1%)	1 (0.5%)	1 (0.5%)
Hypotension	2 (1.1%)	1 (0.5%)	2 (1.1%)	–
Prolonged QTc interval	1 (0.5%)	–	1 (0.5%)	–
Palpitations	5 (2.8%)	2 (1.1%)	1 (0.5%)	–
Dermatological				–
Pruritus	6 (3.3%)	2 (1.1%)	1 (0.5%)	–
Rash	1 (0.5%)	–	1 (0.5%)	–
Sweating	5 (2.8%)	4 (2.2%)	–	–
Other				
Any infection	4 (2.2%)	2 (1.1%)	–	–

Legend: N, number of individuals in the analysis; SSRI, selective serotonin reuptake inhibitor.

Secondary outcomes are reported in Table 2. Overall, no differences between vortioxetine and SSRIs emerged in terms of safety and tolerability outcomes, including discontinuation due to any cause, ASEC total score at different timepoints, and ASEC highest score. There were no events of non-suicidal self-injury. One participant in the vortioxetine arm attempted suicide, and one participant in the SSRI arm died by suicide. Given the low frequency of these events, differences between arms were not estimated. There were also relatively few deaths and serious adverse events, preventing an accurate risk estimation. With regards to efficacy outcomes, no differences between vortioxetine and SSRIs emerged in terms of number of responders (MADRS total score reduction of at least 50%), mean MADRS score, and mean EQ-5D score at different timepoints, according both to the LOCF and the multiple imputation analysis. Lastly, we found no significant differences in terms of mean SBT scores at 1 and 3 months, but the difference was statistically significant in favour of SSRIs at 6 months in the LOCF (N = 356; estimate 1.14; 95% CI 0.21–2.06; p = 0.016), also after adjustment (N = 337; estimate 1.24; 95% CI 0.26–2.22; p = 0.014), while the multiple imputation analysis did not provide statistically significant results. These results did not change after removing participants diagnosed with dementia (Supplement E).

## Discussion

To the best of our knowledge, this is by far the largest randomised, pragmatic clinical trial primarily comparing the tolerability of vortioxetine vs. commonly prescribed SSRIs in a population of older adults. As for the primary outcome, we found a significant difference in favour of SSRIs. The magnitude of this difference was relatively small (p = 0.042) and secondary analyses on the primary outcome did not confirm an advantage of SSRIs over vortioxetine. These results do not support the hypothesis that vortioxetine is superior to SSRIs in terms of treatment discontinuation due to adverse events. Such hypothesis was supported by the distinctive pharmacokinetic and pharmacodynamic properties of vortioxetine, which have suggested that this new generation antidepressant may be particularly suitable for vulnerable populations, such older adults, with tolerability advantages over the SSRIs.^16^^,^^20^^,^^21^ However, the present study failed to show tolerability advantages of vortioxetine over the group of SSRIs. This result was largely supported by secondary analysis, including the per-protocol analysis, mixed model analyses adjusting for several confounders, analyses at different timepoints, as well as the survival analysis considering time to discontinuation due to adverse events. Further, other secondary outcomes exploring undesirable effects of treatments, such as discontinuation due to any cause, serious adverse events, overall mortality, and ASEC mean scores at different timepoints, were all consistent with the primary outcome, while cases of deaths by suicide and non-suicidal self-injury were too few to allow a risk estimate. Overall, in terms of tolerability, point estimates showed that the SSRIs outperformed vortioxetine, although a small significant difference emerged only for the primary analysis. Anxiety/irritability and diarrhoea appeared to be relatively more common and severe in people receiving vortioxetine, although the overall small number of events prevented a precise statistical estimate (Table 3). Overall, considering the study sample size and statistical power, as well as its pragmatic fashion and real-world generalisability, these results provide important evidence on the absence of tolerability advantages of vortioxetine over the SSRIs in older adults. This contradicts what has been repeatedly hypothesised in existing literature reviews^21^^,^^27^ and clinical guidelines,^31^^,^^53^ which are however largely based on pre-clinical pharmacological data^21^ and inconclusive indirect estimates derived from placebo-controlled studies.^24^^,^^27^

Vortioxetine and SSRIs appeared to be similar in terms of beneficial outcomes, with the only exception of cognitive profile, as individuals on SSRIs showed a SBT mean score of about one point higher than those on vortioxetine at the end of the study. This difference might be of clinical relevance, considering that study participants had an SBT mean baseline score of about 4.5 on a scale ranging from 0 to 28, where 5 is the cut-off for “questionable impairment” and 10 for “impairment consistent with dementia”. The significant benefit remained but the strength of association was attenuated after adjusting for confounders. Surprisingly, this result is not consistent with available data suggesting the choice of vortioxetine for individuals with depression-related cognitive symptoms.^54^ However, these findings should be interpreted with caution, as the SBT is a simple tool that can provide a general proxy for cognitive performance, but it is not specifically designed to detect cognitive problems in individuals suffering from depression.

This study has several limitations. First, we chose a highly pragmatic primary outcome (i.e., treatment discontinuation due to adverse events), which could not be assessed under blind conditions. Although this might increase the risk of detection bias, we consider this possibility unlikely, as most participants discontinuing treatment for adverse events did not actually involve the study psychiatrist in this decision. Moreover, data from the ASEC rating scale, administered under blind conditions to measure the number and severity of adverse events, were largely consistent with the primary analysis, and so were other outcomes such as mortality and serious adverse events, not prone to be altered by knowing the treatment allocation. Second, lack of blinding might be associated with a higher risk of performance bias. However, we are confident that this risk was arguably negligible, considering that over 50 psychiatrists were involved in participant recruitment from many different settings in the context of their daily clinical routine. Therefore, it seems unlikely that a relevant proportion of them might have systematically favoured or disfavoured one of the two treatments based on a priori overt or covert opinions or expectations towards vortioxetine or SSRIs, which are both already marketed for depression in Italy. Further, we found that throughout follow-up there were no significant differences between the two arms in terms of antidepressants’ dose prescribed, additional psychotropic medications, and/or additional care including psychological support. Third, we chose to group SSRIs together because previous research has shown similar efficacy and tolerability profiles for these medications.^8^^,^^38^ Nevertheless, potential subtle differences in tolerability, which are speculated to be more pronounced in the elderly population, cannot be completely ruled out. For instance, there may be an elevated likelihood of sedation associated with citalopram, escitalopram, and paroxetine, as well as a higher risk of anticholinergic effects specifically linked to paroxetine. Additionally, distinct patterns of interactions with concomitant medications cannot be completely ruled out and may have played a role in the overall tolerability observed. Moreover, fluoxetine and fluvoxamine were prescribed to a limited number of participants (four and one, respectively), making it challenging to extrapolate the findings to a broader population. This may mirror typical clinical practices, as these medications are seldom infrequently prescribed to older individuals, mostly due to the risk of pharmacological interactions associated with fluvoxamine and the prolonged half-life of fluoxetine. Fourth, 20–25% of participants did not complete follow-up assessments for various reasons. However, this unlikely affected the quality of the analysis, considering the similar distribution between groups of participants lost at follow-up, the statistical power allowed by study completers, and the consistent findings of per-protocol and primary analyses. Fifth, we did not assess anxiety symptoms at baseline, which could be a confounding factor, as such symptoms are often mistaken for adverse drug reactions and may lead to treatment discontinuation. However, the number of people with moderate-to-severe “inner tension” (as defined by a score of ≥3 on the item 3 of the MADRS), which can be a proxy for anxiety symptoms, did not differ between the two arms (Table 1). In addition, GLMM analyses adjusting for the covariate “comorbid psychiatric disorders” (largely represented by anxiety conditions) were broadly consistent with the main analyses. Sixth, the vortioxetine arm included a slightly higher proportion of individuals with two or more medical comorbidities (30.2% vs. 24.2%), which may have affected the outcomes of interest; however, the overall medical burden (as assessed by the CACI) was largely comparable between the two arms, and adjusted analyses including the CACI score were in line with the main analyses. Finally, despite being no differences in terms of overall tolerability, differences in terms of specific adverse events cannot be excluded, considering that the relatively low number of individual adverse events prevented an accurate estimate of the difference between arms. Further, we were not able to detect some of the side effects occurring in older adults after relatively long periods of treatment, such as hyponatraemia or sexual dysfunctions, for which vortioxetine showed prosing results in a previous RCT.^55^

In conclusion, our findings have important implications for clinical practice, research, and policy. Psychiatrists should be aware that vortioxetine is a valid option for the treatment of MDD in older adults, although it is unlikely that it may provide additional benefits in terms of tolerability as compared to commonly prescribed SSRIs. Particularly, gastrointestinal symptoms are possibly more common and severe for vortioxetine than SSRIs, an issue which might be addressed by slower titration (e.g., using drops formulation). Current guidelines should acknowledge these results by de-emphasising the promising role of vortioxetine in older adults in light of its better tolerability. Future large, observational, registry-based studies might shed light on the tolerability of vortioxetine compared to SSRIs in terms of relatively uncommon but potentially serious adverse events, such as hyponatraemia, falls, bleeding, and arrhythmias.

The overall efficacy of vortioxetine proved to be comparable to that of SSRIs, with the possible exception of depression-related cognitive dysfunction, where SSRIs appeared more effective. Future experimental research specifically focusing on this symptomatic dimension is needed to ultimately clarify the possible role of vortioxetine, considering heterogeneous results from current evidence.

## Contributors

G.O., C.G., A.B., B.D., and C.B. conceptualised and designed the study; G.O., C.G., and C.B. had leading roles of funding acquisition, project administration, supervision and validation of data collection, and data analysis; I.M. had roles of software design for data collection and management; M.T. and M.C. performed the statistical analyses; A.B. and B.D. supervised the activities of data management and statistical analysis; A.A., F.B., C.C., G.C., R.C., A.D., P.D., G.M., R.R., E.A., L.T. coordinated and supervised the recruiting and administrative activities of their local recruiting centres; G.O., C.G., and C.B. wrote the original draft of the paper with reviews and edits from all the other authors. All authors had full access to all the data in the study and accept responsibility for the decision to submit for publication.

## Data sharing statement

The dataset containing all de-identified individual participant data used for the analyses has been uploaded in the online repository EUDAT B2Share under the license “Creative Commons Attribution-NonCommercial-NoDerivs” (CC-BY-NC-ND). Researchers interested in the analysis of such data will be asked to forward a formal request to the VESPA Study Committee, composed by G.O., C.B., C.G., A.B., B.D. The request must include details about the research hypothesis, the variables required, and the statistical methodology that will be employed. The VESPA Study Committee will determine whether to accept the request, and if approved, a data access agreement will be created and signed by both parties.

## Declaration of interests

A. A. received honoraria for presentations from Lundbeck, Viatris, Angelini. E. A. received research grants, consulting fees and honoraria for presentations from Allergan, Angelini, Doc Generici, FB-Health, Janssen-Cilag, Lundbeck, Otsuka, Fidia, Recordati. E. A. C. received support for attending meetings from Janssen-Cilag. F. B. received consulting fees and support for attending meetings from Angelini, Janssen-Cilag, Otsuka. G.M. received research grants, consulting fees and honoraria for presentations from Angelini, Doc Generici, Janssen-Cilag, Lundbeck, Neuraxpharm, Otsuka, Pfizer, Servier, Rovi, Recordati. R. d. F. received consulting fees and honoraria for presentations from Janssen-Cilag, and support for attending meetings from Janssen-Cilag and ROVI. All the other authors have no competing interests to disclose.

## Appendix A Supplementary data

Supplemental Material

Summary Italian
