# Supplementary material for: Tolerability of vortioxetine compared to selective serotonin reuptake inhibitors in older adults with major depressive disorder (VESPA): a randomised, assessor-blinded and statistician-blinded, multicentre, superiority trial
Source: eClinicalMedicine. 2024 Feb 15;69:102491. doi: 10.1016/j.eclinm.2024.102491 (PMC10879669; doi:10.1016/j.eclinm.2024.102491)
Supplement: Supplemental Material [file mmc1.pdf]

Supplemental material

Ostuzzi et al. Tolerability of vortioxetine compared to selective serotonin reuptake inhibitors in older adults with major depressive disorder (VESPA): a randomised, assessor-blinded and statistician-blinded, multicentre, superiority trial

Index

A. Original protocol 2

B. Participant information sheet *[Italian]* 20

C. Informed consent form *[Italian]* 26

D. Approval by the Ethics Committee of the Coordinating Centre 27

E. Deviations from the original protocol and rationale 28

F. Assessment of pragmatism with the tool PRECIS-2 30

G. Antidepressants' mean dose and additional treatments 32

H. List of investigators of the VESPA Study Group 33

Supplement A. Original protocol

Assessing tolerability and efficacy of vortioxetine versus SSRIs in elderly patients with depression: a pragmatic, multicenter, open-label, parallel-group, superiority, randomized trial (study protocol version 1.5)

Principal investigator: Prof. Corrado Barbui
Azienda Ospedaliera Universitaria Integrata (AOUI) Verona - Unit of Psychiatry
WHO Collaborating Centre for Research and Training in Mental Health and Service Evaluation
Department of Neuroscience, Biomedicine and Movement Sciences
Section of Psychiatry, University of Verona, Verona, Italy
Acronym: VESPA (Vortioxetine in the Elderly vs. SSRIs: a Pragmatic Assessment)
Funding: Agenzia Italiana del Farmaco (AIFA); Bando per la Ricerca Indipendente 2016; code: 2016-0234923
The study sponsor has no role in study design; collection, management, analysis, and interpretation of data; writing of the report; and the decision to submit the report for publication. The study sponsor had no ultimate authority over any of the listed activities.

List of abbreviations

ASEC	Antidepressant Side-Effect Checklist
CACI	Charlson Age-Comorbidity Index
ECG	Electrocardiogram
EMA	European Medicines Agency
EQ-5D	EuroQual 5 Dimensions
EAB	Ethics Advisory Board
FDA	Food and Drug Administration
FUF	Follow-Up Form
GABA	Gamma-Aminobutyric Acid
GDT	Global Burden of Disease
ICH	International Council for Harmonisation of Technical Requirements for Pharmaceuticals for Human Use
ITT	Intention to Treat
LOCF	Last Observation Carried Forward
MADRS	Montgomery–Åsberg Depression Rating Scale
Mg	Milligrams
NICE	National Institute for Health and Care Excellence
OU	Operative Unit
QTc	Corrected Q-T interval
RF	Recruitment Form
SBT	Short Blessed Test
SNRI	Serotonin and Norepinephrine Reuptake Inhibitor
SSN	Sistema Sanitario Nazionale
SSRI	Selective Serotonin Reuptake Inhibitor
TCA	Tricyclic antidepressant
WHO	World Health Organization

Executive summary

Background. Depression is a highly frequent condition in the elderly, with a huge impact on quality of life, life expectancy, and medical outcomes. This condition has also indirect costs related to the psychological and economic burden on family members, caregivers, and the wider society. SSRIs are the most commonly prescribed agents in elderly depressed patients and, although generally safe, they may be associated with tolerability issues. Vortioxetine is an

antidepressants with a novel mechanism of action. Based on available studies, this drug has a promising tolerability profile in the elderly, as it does not adversely affect psychomotor or cognitive performance, wakefulness, body weight, and electrocardiogram parameters.

Objectives. Assessing the comparative tolerability, safety and efficacy of vortioxetine compared with the SSRIs as a group (including sertraline, citalopram, escitalopram, paroxetine, fluoxetine, fluvoxamine) in elderly patients affected by major depression. The primary outcome will be the withdrawal rate due to adverse events. Secondary outcomes will include: mortality, suicide, self-harm, occurrence of any adverse event. Validated rating scales will be administer by blind staff to assess efficacy, tolerability, quality of life, and the severity of medical comorbidities.

Methods. This is a pragmatic, multicenter, open-label, parallel-group, superiority, randomized trial. Fourteen Italian Community Psychiatric Services will consecutively enroll elderly patients suffering from an episode of major depression who get in contact over a period of 12 months. By employing web-based application, doctors will (a) quickly randomize patients to vortioxetine or one of the SSRIs, chosen on the basis of clinical judgment; (b) collect basic socio-demographic and clinical data through a Recruitment Form. Trained assessors blinded to treatment allocation will administer five validated rating scales: Montgomery–Åsberg Depression Rating Scale (MADRS), Antidepressant Side-Effect Checklist (ASEC), EuroQual 5 Dimensions (EQ-5D), Charlson Age-Comorbidity Index (CACI), and Short Blessed Test (SBT). Patients will be followed-up and assessed after 1, 3 and 6 months with the same scales and a Follow-up Form.

Expected results. On the basis of current literature, we hypothesize vortioxetine to be superior to SSRIs as a group in terms of tolerability. As we expect vortioxetine to reduce the withdrawal rates due to adverse events of about 12% compared to SSRIs, and assuming that about 23% of the participants could be lost within 6 months, we aim at enrolling 358 patients (179 in each group).

Background

Depression is among the most disabling conditions worldwide (GBD 2015). Depression occurs in up to 4.5% of elderly people in community settings (Eden et al., 2012) and up to 40% in residential homes and hospitals (Ames 1990; Blazer et al., 2001). In elderly people, depression is associated with poor quality of life, reduced life expectancy, high risk of suicide (Heisel et al., 2006), high risk of cognitive decline and dementia (Steenland et al., 2012), reduced adherence to medical treatments and, therefore, poorer medical outcomes as compared with adult populations (Jiang et al., 2001). Depression in the elderly is also associated with psychological and economic burden on family members, caregivers, and the wider society.

In the general population of individuals with depression, SSRIs are considered effective and safe (Cipriani et al., 2018). In elderly patients with depression, SSRIs are considered effective and generally safer compared to other classes of antidepressants (Mottram, 2016), although this age group may be particularly vulnerable to adverse events due to aging itself, medical comorbidities, multiple treatments, and high risk of pharmacological interactions. The most common adverse events associated with SSRIs in the elderly include hyponatraemia, postural hypotension, falls, gastrointestinal bleeding, and sexual dysfunctions. Some authors hypothesized that cognitive impairment may be favoured by the anticholinergic profile of some antidepressants, for example paroxetine (Bali et al. 2016). Clinically useful alternatives to the SSRIs are lacking, considering that tricyclic antidepressants (TCAs), Serotonin and Norepinephrine Reuptake Inhibitor (SNRIs) and mirtazapine are at higher risk of a number of adverse events, including sedation, confusion, urinary retention, cardiovascular and gastrointestinal issues.

Vortioxetine is a novel antidepressant, licensed for the treatment of depression in 2013 by FDA and EMA. Its mechanism of action is not fully understood, however, despite similarities with SSRIs, its pharmacodynamic profile is claimed to be

novel, and it is classified among “other antidepressants” by the World Health Organization (WHO). Therefore, based on this new pharmacological profile, vortioxetine may represent an alternative to existing antidepressant drugs (EMA, 2014a). A recent Cochrane systematic review and meta-analysis (Koesters et al., 2017) retrieved a total of 15 randomized trials on vortioxetine for the treatment of depression (7746 participants). The analysis showed vortioxetine to be effective compared to placebo. No statistically significant differences emerged between vortioxetine and SNRIs as a class, in terms of both efficacy and tolerability. Interestingly, no randomized trials compared vortioxetine to SSRIs or other antidepressants. Therefore, the comparative tolerability of this drug still needs to be fully addressed. Additionally, data on selected populations, including the elderly, are lacking, with the exception of one trial showing that vortioxetine may be more effective than placebo in terms of response (n=301, RR 1.49, 95% CI 1.14 to 1.95) (Katona et al., 2012). However, this study found no statistically significant differences in terms of overall acceptability (withdrawals due to any cause) and tolerability (withdrawals due to adverse events). Compared to duloxetine, no differences emerged in terms of efficacy, acceptability and tolerability.

Many authors claimed a potentially safer tolerability profile of vortioxetine compared to other antidepressants, including SSRIs, as vortioxetine has similar pharmacokinetic properties in young and older adults (FDA, 2013), and does not adversely affect psychomotor or cognitive performance, wakefulness, body weight, and electrocardiogram parameters (McIntyre et al., 2014; Kelliny et al., 2015). Further, possible beneficial effects on cognition emerged from three randomized trials in patients with cognitive impairment (Miskowiakn et al., 2016), but this outcome dimension needs more detailed investigation.

Against this background, this study aims at assessing the comparative tolerability, safety and efficacy of vortioxetine for the treatment of depression in elderly patients by employing a randomized, open-label design. According to a pragmatic principle, vortioxetine will be compared to the SSRIs as a group. In the SSRI group, doctors will be free to make the most appropriate choice based on clinical judgment. The primary outcome will be treatment tolerability, pragmatically measured as withdrawal rate due to adverse events. We expect that this study will generate evidence with direct clinical and policy implications. Clinically, it will provide doctors with usable information on the beneficial and harmful consequences of the use of vortioxetine in elderly depressive patients, that currently remain an “orphan” population with almost no evidence base; in terms of policy, it will inform current regulatory debate, as well as guideline development, on which antidepressant should be used as first-line treatment in this age group.

Objectives

Primary objective. The present study will assess if vortioxetine is better tolerated as compared to the SSRIs as a group in elderly patients suffering from a current major depressive episode. Tolerability will be measured as the number of patients withdrawing from allocated treatment due to adverse events at the end of the study (6 months).

Secondary objectives. In addition, the VESPA study will assess a number of secondary tolerability outcomes, including acceptability (number of withdrawals from allocated treatment due to any cause, endpoint 1), overall mortality (endpoint 2), self-harm episodes (endpoint 3), mortality by suicide (endpoint 4), occurrence and severity of adverse events (endpoint 5), number of responders (endpoint 6), efficacy (endpoint 7), quality of life (endpoint 8), cognitive performance (the last three outcomes will be measures as the mean change score on a validated rating scale, endpoint 9).

Study design

Synthetic overview. The study group is composed of twelve Italian Psychiatric Services plus the Mario Negri Institute for Pharmacological Research, a not-for-profit biomedical research organization with extensive experience in conducting

randomized studies. Doctors working in Psychiatric Services will consecutively enrol elderly patients suffering from an episode of major depression and requiring treatment with an antidepressant who are in contact over a period of 12 months, including also first episode patients. Patient enrolment will be performed as part of routine clinical practice, irrespective of the setting of care (inpatients and outpatients). A list of all the excluded patients among the eligible ones will be completed by each doctor. After having explained to the eligible patients the main features of the study, and having received a signed informed consent, doctors will be able to include and randomize patients by employing a web-based tool, accessible from tablet and PC, which will allow to allocate patients according to a concealed, computer-generated randomization list. In order to safeguard the allocation concealment, patients' main socio-demographic and clinical data at baseline will be entered into the web-based tool before random allocation. After random allocation, patients will be prescribed vortioxetine or one of the SSRIs following usual care. The choice of the SSRI will be based on clinical judgment, according to a shared decision-making process. Before random allocation, three validated rating scales for assessing depressive symptoms, quality of life, and medical comorbidity, will be administered by trained assessors. No blinding to treatment allocation will be applied for patients and doctors. Patients will be assessed at 1, 3 and 6 months with the three previous rating scales, and one further rating scale measuring adverse events related to the antidepressant. The end of the study is defined as the date of the last visit of the last patient enrolled in the last recruiting centre. At the end of the study, statistical analysis will be performed by a bio-statistician blinded to treatment allocation.

Inclusion criteria. The following inclusion criteria will be adopted:

a. 65 years old or above;
b. willing to participate by signing an informed consent;
c. suffering from an episode of major depression, based on clinical judgment (guided by DSM-5 criteria);
d. treatment with an antidepressant is clinically appropriate, based on clinical/medical judgment;
e. agreement between investigator and patient to discontinue any current antidepressant, second generation antipsychotic, or lithium. According to the pragmatic design of the study, the discontinuation will be performed according to common routine practice. No specific protocols for discontinuation will be applied. All other concomitant medications are allowed;
f. uncertainty about which trial treatment would be best for the participant.

Exclusion criteria. The following exclusion criteria will be adopted:

a. dementia, of any type and stage, as formally diagnosed by a specialist (geriatrician, neurologist, or others);
b. diagnosis of schizophrenia or bipolar disorder;
c. clinical conditions or treatments which contraindicate the use of oral vortioxetine or SSRIs, according to clinical/medical judgment (for example conditions or treatments at high risk of bleeding, convulsions, serotoninergic syndrome, hyponatraemia, etc.). All concomitant medications will be prescribed according to routine clinical practice, in compliance with the synthesis of the product characteristics (*Riassunto delle Caratteristiche del Prodotto – RCP*) registered in the databank of the AIFA (*Agenzia Italiana del Farmaco*) (available at https://farmaci.agenziafarmaco.gov.it/bancadatifarmaci/cerca-farmaco).

No exclusion criteria will be applied in terms of setting of recruitment, severity of depression, past use of psychotropic drugs, current use of benzodiazepines (as long as RCP indications are respected), number and severity of medical comorbidities, and multiple pharmacotherapy.

Treatments

Vortioxetine and SSRIs are currently marketed in Italy, and they are both indicated for the treatment of major depression. Only formulations reimbursed by the SSN (Sistema Sanitario Nazionale) will be used in the VESPA study. Patients allocated to vortioxetine will take an oral starting dose of 5 milligrams/day. Vortioxetine is marketed both as coated tablets (of 5, 10, 15, 20 mg) or drops (20 mg/mL; 1 drop=1 mg), and the choice between the two formulations will be based on clinical judgment and patient preferences. A flexible dosing schedule will be applied. Doctors will be free of increasing or decreasing the dose according to patient clinical status, within the licensed dose range. According to the technical specifications of vortioxetine, approved by the EMA, 5 mg/day will represent the lower effective dose, and doses above 10 mg/day should be administered cautiously, considering the lack of clinical data on the tolerability of this drug in the elderly (EMA 2014a). All dose changes will be recorded. Following randomization, treatment will be taken daily for six months. This is in line with current evidence suggesting a length of treatment of at least 9-12 months after recovery (WHO, 2012). At the end of the follow-up period, the choice of continuing or withdrawing the treatment will be based on clinical judgment, according to ordinary practice.

Experimental drug. Vortioxetine is an antagonist to 5-HT3, 5-HT1D and 5-HT7 receptors, a partial agonist to the 5-HT1B receptor and a 5-HT1A receptor agonist (EMA 2014b). Therefore, its pharmacological mechanism is likely to be related with both a direct modulation of the serotoninergic receptor activity and inhibition of the serotonin transporter, although this is not fully understood yet. It is hypothesized that serotonin transporter inhibition combined with the several other actions of vortioxetine at 5HT receptors, mainly at 5-HT3 receptor, enhances not only the release of serotonin, but also modulates the release of other neurotransmitters within various brain circuits (enhanced release of norepinephrine, dopamine, histamine, acetylcholine, and glutamate; reduced gamma-aminobutyric acid (GABA) signaling. These actions could improve the efficiency of information processing in malfunctioning brain circuits by facilitating long-term potentiation, neuroplasticity, and increased firing of pyramidal neurons (Du Jardin et al., 2016, Pehrson et al., 2016). By affecting the levels of neurotransmitters involved in cognitive processes, vortioxetine seems to have a beneficial effect on cognition, as showed in preclinical studies (Pehrson et al. 2014; Sanchez et al. 2015) and in randomized trials in adults and elderly with depression (Katona et al. 2012; McIntyre et al. 2014). The pharmaceutical profile of this new antidepressant may be particularly favourable for elderly patients with depression, as its novel mechanism of action could underpin an enhanced tolerability profile, and also a favourable effect on neurocognitive performances.

Besides the potential advantages highlighted above, some risks should be taken into account. Although available data show an overall safe and tolerable profile of vortioxetine in both adults (Koesters et al., 2017) and elderly patients (Katona et al., 2012; McIntyre et al., 2014; Kelliny et al., 2015), a higher occurrence of rare but severe events (such as bleeding, seizures, suicide or self-harm) cannot be excluded a priori, considering the relatively few old patients included in these study and the relatively short periods of follow-up. According to the Summary of Product Characteristics (SPC) vortioxetine may be burdened by the following adverse events: abnormal dreams, dizziness, diarrhoea, constipation, vomiting, pruritus are considered common (ranging from 1 in 100 patients to 1 in 10 patients), while nausea is very common (frequency greater than 1 patient in 10). Furthermore, although vortioxetine showed to be effective over placebo, with response rates similar to those emerged from SSRIs trials in the elderly (around 50%, see Mottram et al., 2006, and Koesters et al., 2017), available efficacy data of vortioxetine compared to venlafaxine e duloxetine are inconclusive, and can hardly be extended to SSRIs (Koesters et al., 2017). Therefore, also the possibility of a low efficacy cannot be excluded a priori.

Comparator. The comparator will be represented by any antidepressant of the class of serotonin selective reuptake inhibitors (SSRIs). SSRIs are commonly prescribed in the elderly, as they have been shown to be safer than tricyclic

antidepressants (TCAs) (Mottram et al., 2006), which are burdened with possible direct cardiovascular effects, as well as anticholinergic effects. Also, SSRIs are generally preferred to SNRIs and other atypical antidepressants, whose use may be limited by the risk of blood pressure alterations (venlafaxine), seizures (bupropion), sedation and falls (mirtazapine) (Wiese 2011; Coupland et al., 2011). SSRIs are suggested as a first-choice treatment for adults as well as older patients, considering their favorable risk-benefit ratio. However, they are not free from risks, particularly in the elder, who may have an increased risk of bleeding, hyponatraemia, postural hypotension, falls and drug interactions (for fluoxetine, fluvoxamine and paroxetine above all) (NICE, 2016). The choice of the SSRI will be based on clinical judgment and shared decision-making process.

For patients allocated to SSRIs, clinical judgment will be used to select which SSRI will be prescribed. SSRIs marketed in Italy include: sertraline, citalopram, escitalopram, paroxetine, fluoxetine, fluvoxamine. The choice of the formulation will be based on clinical judgment and patient preferences. All SSRIs are available as tablets. Citalopram, escitalopram, paroxetine and fluoxetine are also available as drops. A flexible dosing schedule will be applied. Doctors will be free of increasing or decreasing the dose according to patient clinical status, within the licensed dose ranges (as approved by EMA) (EMA 2014a and 2014b). All dose changes will be recorded. Following randomization, treatment is to be taken daily for six months. At the end of the follow-up period, the choice of continuing or withdrawing the treatment will be based on clinical judgment, according to ordinary practice. Routine care outside the trial will be preserved as usual. Concomitant benzodiazepines for reducing anxiety or improve sleep are allowed.

During the study, participants will be seen as often as clinically indicated. Blinded assessments will be performed at one, three and six months, concomitantly to regular visits, scheduled according to usual practice. Considering the pragmatic characteristics of this study, including the use of already marketed antidepressants for licensed indications under unblind conditions, no stopping rules will be applied.

Synthesis of treatments and dosing schedule

**Medication**	**Licensed dose range in the elderly**	**Notes**
vortioxetine	5 – 20 mg/day	The minimum effective dose of 5 mg vortioxetine once daily should always be used as an initial dose for patients aged ≥ 65 years. Caution should be exercised when prescribing to elderly patients at doses above 10 mg vortioxetine once daily.
sertraline	50 – 200 mg/day	
paroxetine	20 – 40 mg/day	
citalopram	10 – 20 mg/day	Half of the dose range prescribed in adults.
escitalopram	5 – 10 mg/day	Half of the dose range prescribed in adults.
fluoxetine	20 – 60 mg/day	Caution is required when the dose is increased in the elderly, and generally the daily dose should not be above 40 mg/day. The maximum recommended dose is 60 mg/day.
fluvoxamine	100 – 300 mg/day	In elderly patients, titration should be slower and the dosage should always be established with caution.

Outcomes

Primary Outcome. Patients withdrawing from allocated treatment due to adverse events at the end of the study (6 months) will be the primary outcome. This is a useful and pragmatic measure of the overall tolerability of a treatment (Barbui et

al., 2008). Six months represent a clinically sound time frame for assessing the overall tolerability of medications, including both acute, short-term effects (nausea, headache, sedation, etc.), and medium-long-term effects (bleeding, hyponatraemia, ECG abnormalities, etc.). Pragmatically, the antidepressant treatment will be considered withdrawn due to adverse effects when the drug is stopped for more than two consecutive weeks following the occurrence of any adverse event, based on clinical judgment. Withdrawals due to adverse events will be also evaluated after 1 and after 3 months from the randomization, in order to describe the trends of this event over time.

Secondary Outcomes

1. Acceptability, measured as the number of withdrawals from allocated treatment due to any cause on the number of randomized patients at 1, 3, 6 months. Pragmatically, the treatment will be considered withdrawn when the assigned treatment is interrupted for two or more consecutive weeks, due to any reason.
2. Overall mortality, measured as the number of deaths on the number of randomized patients at 1, 3, 6 months. This is a pragmatic outcome measure of the overall efficacy and safety of treatments;
3. Self-harm, measured as the number of self-harm episodes on the number of randomized patients at 1, 3, 6 months;
4. Suicide risk, measured as the number of deaths by suicide on the number of randomized patients at 1, 3, 6 months;
5. Adverse events (continuous), measured as the mean change score at the Antidepressant Side-Effect Checklist (ASEC) (Uher et al., 2009) at 1, 3, 6 months. ASEC is a validated rating scale measuring the occurrence and severity of 21 adverse events of antidepressants drugs;
6. Efficacy (dichotomous), measured as the number of responders according to a reduction of at least 50% of the baseline score of the Montgomery–Åsberg Depression Rating Scale (MADRS) at 1, 3, 6 months. MADRS is a validated, ten-item questionnaire for assessing the severity of depression (Montgomery and Åsberg, 1979). This scale is largely employed in clinical practice and in randomized trials on antidepressants, including vortioxetine;
7. Efficacy (continuous). Mean change scores at MADRS at 1, 3, 6 months;
8. Quality of life (continuous). Mean change scores at the EQ-5D at 1, 3, 6 months. EQ-5D is a self-administered validated rating scale for assessing quality of life (EuroQol Group, 1990). This tool explores five areas, including mobility, self-care, usual activities, pain/discomfort, anxiety/depression, and assesses the overall subjective perception of health with an analogic scale. The EQ-5D has been frequently used in randomized trials on antidepressants;
9. Cognitive performance (continuous). Mean change scores at the Short Blessed Scale (SBT) (Katzman et al., 1983) at 1, 3, 6 months. SBT is a validated, six-item weighted instrument, originally designed to identify dementia, which assesses orientation, registration, and attention.

In addition to these tools, the Charlson Age-Comorbidity Index (CACI) will be employed. This is a validated rating scale used to evaluate the degree of medical comorbidity, and to predict the 10-year survival in patients with multiple comorbidities (Charlson et al., 1994). This will allow an adjustment for the level of medical comorbidity in the phase of statistical analysis.

Validated rating scales (ASEC, MADRS, EQ-5D, CACI, SBT) will be administered by blinded outcome assessors. Laboratory exams and ECG will be prescribed only if clinically indicated, according to routine clinical practice. Hyponatraemia and QTc prolongation will be registered by the time of their occurrence.

The main analysis will be focused on endpoints measured at the end of the study (6 months). Data from 1 and 3 months will be employed only on a descriptive fashion, in order to describe the trends of the events of interest over time, and the onset time of action of study medications.

All the information reported on the follow-up form refer to the period elapsed from the previous visit. With regards to rating scales, the ASEC refers to the period elapsed from the previous visit, the MADRS refers to the previous month, the CACI, the SBT, and the EQ-5D refer to current time.

Medication interruption. Patients might interrupt the treatment for many reasons, related for instance to their own perception of the study medication, to other co-occurring conditions, to the clinician's evaluation, or to several external factors. For the aims of the VESPA study, we will register reasons for interruption of the medication in simple categories, as follows:

a. Occurrence of a medical conditions and/or beginning of a medical treatment no longer compatible with the use of vortioxetine or SSRIs;
b. Occurrence of a psychiatric conditions and/or beginning of psychiatric treatment no longer compatible with the use of vortioxetine or SSRIs;
c. Ineffectiveness of the study medication on depressive symptomatology;
d. Adverse events attributable to the study medication. In this case, the most relevant side effect responsible for the suspension (and its severity) will be registered.

The clinician will choose among the above reported categories according to the overall clinical evaluation, in line with routine clinical practice.

Study interruption. According to the ITT principle, all randomized patients will be followed until the end of the study. The patient will withdraw the study only in case of:

- Withdrawal of the informed consent to participate to the study;
- Death. In this case, all available information until the data of death will be collected;
- The patient is unreachable or cannot attend the follow-up visits (e.g. relocation in a different city).

Methods

Randomization. Patients will be randomly assigned to one of the two treatment groups (vortioxetine or SSRIs) with an equal probability of assignment to each treatment (allocation ratio 1:1). A centralized randomization procedure will be employed. The trial biostatistician will prepare the sequence of treatments randomly permuted in blocks of constant size. The site investigators will not know the block size. The allocation will be stratified by recruiting center. Recruiting doctors will use an online, web-based application that will provide, after basic information on the enrolled participant is entered, the patient's identification number (ID) and the allocated treatment.

Blinding. In agreement with a pragmatic principle, an open-label approach will be employed. This type of design has the advantage of preserving ordinary clinical routine, although it is associated with a higher risk of performance and detection bias. While performance bias is strongly related to positive or negative expectations of doctors towards medications, and can hardly be controlled, the risk of detection bias will be minimized by (a) employing a hard outcome, unlikely to be altered by the lack of blinding, as primary outcome, and (b) providing a blinded assessment of complex measures, such as validated rating scales.

Data collection and management. Patients will be recruited in Italy. Psychiatric Services will be asked to recruit consecutive patients meeting the inclusion/exclusion criteria over a 12-month period. Before entering the study, patients

will be asked to provide oral and written informed consent to participate. Clinical and demographic characteristics will be collected at baseline, before randomization, using the recruited form (RF) included in the electronic application, which will include:

a) socio-demographic information: date of birth, sex, nationality, housing conditions, marital status, educational level, working conditions;
b) clinical information: year of first contact with a psychiatrist, psychiatric diagnosis (including use of alcohol or other substances), previous hospitalizations in Psychiatric wards, previous episodes of self-harm, concomitant medical conditions, current and previous psychotropic medications, current medical treatments.

Five validated rating scales (MADRS, EQ-5D, CACI, SBT) will be administered at baseline by staff blinded to patient allocation. Follow-up data will be obtained at 1 month, 3 months and 6 months, using a Follow-Up Form (FUF) included in the electronic application. The FUF will include: outcome measures (possible interruption of the treatment and reason), any update of psychiatric diagnosis, use of alcohol or other substances, new non-pharmacological psychiatric treatments, newly diagnosed medical conditions and medical treatments, hospitalizations in Psychiatric wards, episodes of self-harm. MADRS, EQ-5D, CACI, SBT and ASEC will be administered at each time point under blind conditions by trained staff. All study data collected with the electronic tool will be stored by the IRCCS Istituto di Ricerche Farmacologiche Mario Negri (Milan). The use of digital RF and FUF will optimise data completeness and consistency, as these will be developed with the intent of guiding the procedure of data collection, perform an immediate data validation, in order to prevent mistakes and typos, and allowing doctors to dedicate the minimum amount of time to this procedure, which deviates from ordinary practice. In addition, a set of electronic and manual edit checks will be performed. The statistical analysis will be performed by a bio-statistician blinded to patient allocation of at the Mario Negri Institute for Pharmacological Research. The IRCCS Istituto di Ricerche Farmacologiche Mario Negri is a not-for-profit biomedical research organization, with research programs spanning from molecular to clinical level, with a longstanding experience in many fields of medicine, including mental illnesses.

Although the web-based tool will allow to minimize hard copy documents, some is still needed, in particular: (a) the signed informed consent, and (b) the self-administered rating scales, which will be filled by the patient and immediately entered in the electronic tool by the investigator. The local coordinator of each recruiting centre will have the duty of store and preserve the hard copy documents in a safe, key-locked room or cabinet drawer, accessible only by him/her. File will be preserved for at least 7 years after the end of the study, according to the Italian legislation, (art. 18 d.lg. n. 200/2007; d.lg. n. 219/2006, all. 1, punto 5.2, lett. c) and then destroyed.

Safety. The VESPA study will operatively employ the definitions endorsed by the EC Directive 2001/20/EC (available at: https://eur-lex.europa.eu/LexUriServ/LexUriServ.do?uri=OJ:L:2001:121:0034:0044:en:PDF):

- Adverse Events (AE): any untoward medical occurrence in a patient or clinical trial subject administered a medicinal product and which does not necessarily have a causal relationship with this treatment;
- Adverse Reaction (AR): all untoward and unintended responses to an investigational medicinal product related to any dose administered;
- Serious Adverse Event (SAE) or Serious Adverse Reaction (SAR): any untoward medical occurrence or effect that at any dose results in death, is life-threatening, requires hospitalisation or prolongation of existing hospitalisation, results in persistent or significant disability or incapacity, or is a congenital anomaly or birth defect;
- Unexpected Adverse Reaction (UAR): an adverse reaction, the nature or severity of which is not consistent with the applicable product information (specifically, for the aims of the VESPA study, the RCP from the AIFA

register).

- Suspected Unexpected Serious Adverse Reaction (SUSAR) is any UAR that at any dose:
  - results in death;
  - is life threatening (i.e. the subject was at risk of death at the time of the event; it does not refer to an event which hypothetically might have caused death if it were more severe);
  - requires hospitalisation or prolongation of existing hospitalisation;
  - results in persistent or significant disability or incapacity;
  - is a congenital anomaly or birth defect.

The RCP must be regarded as the primary reference for the common expected adverse events of study medications.
All AE reported spontaneously by the participants or observed by doctors or outcome assessors will be identified, recorded and reported according to SOP #1 (see Appendix). As soon as a serious adverse event occurs, an *ad hoc* form for Serious Adverse Events (SAE) must be filled and forwarded to the Pharmacology Service, University of Verona, in accordance with the EU legislation about pharmacovigilance in clinical research (see http://www.agenziafarmaco.gov.it/content/la-legislazione-di-farmacovigilanza). Operative instructions for registering and reporting SAE is reported in the SOP #1. The severity of adverse events will be registered according to the grades described in Common Terminology Criteria for Adverse Events (CTCAE) Version 5.0 (available at https://ctep.cancer.gov/protocoldevelopment/electronic_applications/ctc.htm#ctc_50):

- Grade 1: Mild, asymptomatic or mild symptoms; clinical or diagnostic observations only; intervention not indicated.
- Grade 2: Moderate, minimal, local or non invasive intervention indicated; limiting age appropriate instrumental ADL[1].
- Grade 3: Severe or medically significant but not immediately life-threatening; hospitalization or prolongation of hospitalization indicated; disabling; limiting self care ADL[2].
- Grade 4: Life-threatening consequences; urgent intervention indicated.
- Grade 5 Death related to AE.

In addition to that, all adverse events potentially related to the antidepressant will be recorded in detail by administering the ASEC at each follow-up visit. The ASEC includes a judgment on the likelihood that the symptom is an adverse event of the antidepressant from the patient's point of view. The site Principal Investigator will inform trial participants and those bodies providing ethical oversight if anything occurs on the basis of which it appears that the disadvantages of participation may be significantly greater than was foreseen.

Sample size and power calculations. On the basis of a Cochrane review comparing different classes of antidepressants in the elderly (Mottram et al., 2006), it is hypothesized that the SSRIs groups will show a withdrawal proportion due to adverse events of about 17% at 6 months (primary study endpoint). On the basis of the results of the vortioxetine randomized trials available in the elderly with (Mahableshwarkar et al., 2015) and without cognitive dysfunction (McIntyre et al., 2015; Katona et al., 2012), it is hypothesized that the proportion of withdrawal due to adverse events within 6 months will be about 5% in the group treated with vortioxetine. Hence, we expect the vortioxetine group to show a clinically significant advantage by reducing the withdrawal rates due to adverse events of about 12%. A sample size of 276 patients (138 in each group) achieves 90% power to detect a difference of 13% between the two withdrawal proportions in favor of vortioxetine. The test statistic will be the two-sided Z test with pooled variance. The significance

[1] Instrumental ADL refer to preparing meals, shopping for groceries or clothes, using the telephone, managing money, etc.
[2] Self care ADL refer to bathing, dressing and undressing, feeding self, using the toilet, taking medications, and not bedridden.

level of the test is targeted at 5%. On the basis of above mentioned studies we can assume that about 23% of the participants could be lost within 6 months (the mean of the total dropout rates of vortioxetine and SSRI studies in the elderly), or could not provide valid data at month 6. Therefore 358 patients (179 in each group) will be enrolled in order to obtain at least 276 evaluable subjects. The sample size calculation has been performed according to Pocock (1983) (online application available at: http://www.sealedenvelope.com/power/binary-superiority/).

Statistical Analysis. The trial biostatistician will be blinded to the treatment groups until the analysis has been completed. Moreover, the trial biostatistician will not be involved in determining patients' eligibility, in administering the treatment, in measuring the outcomes or in entering data.

The Intention to Treat (ITT) population will consist of all randomized patients. This ITT population will be used for the analysis of both primary and secondary outcomes. When possible, in addition to the primary analysis, appropriate statistical methods will adjust for the potential confounding effect of prognostic factors (sex, age, living condition, severity of comorbid medical conditions, previous psychiatric history, MADRS score at baseline).

Maximum effort will be made to retrieve, at the final visit, patients lost at follow-up. Subjects without evaluation at last visit will be treated as failures for primary outcome. Missing values in rating scales will be imputed using the Last Observation Carried forward (LOCF) approach: ratings will be carried forward from the last available assessment to the 6-month follow-up assessment. As a secondary analysis, missing values in rating scales will be imputed following a multiple imputation approach.

In order to check the consistency of the ITT approach, the primary outcome will also be analysed using a per-protocol (PP) approach. According to the PP approach, patients that will discontinue the treatment during follow-up will contribute to the analysis of the outcome only for the time between random allocation and treatment discontinuation (censoring). The analysis of the PP population will be used for confirmatory purposes only.

Analysis of the primary outcome. The proportion of patients withdrawing the study due to adverse events within 6 months of follow-up will be compared between the two groups of treatment using a logistic regression with center (random variable) as a covariate. A multivariable analysis (secondary analysis) will be performed through a Poisson regression model with a robust error variance, given that this procedure allows to estimate relative risks directly (Zou 2004).

Analysis of secondary outcomes. Secondary outcomes will include the following:

a) Dichotomous outcomes occurring within 6 months:
    - Withdrawals due to any cause;
    - Overall mortality;
    - Mortality by suicide;
    - Occurrence of self-harm;
    - Occurrence of adverse events (any), falls, bleeding (any site), hyponatraemia, QTc prolongation;
    - Responders according to the MADRS.

The proportion of patients with the occurrence of the outcome within 6 months will be compared between the two groups of treatment using a logistic regression with center (random variable) as a covariate. When possible, a multivariable analysis will be performed through a Poisson regression model with a robust error variance.

b) Continuous outcomes after 6 months:
    - ASEC;
    - MADRS;
    - EQ-5D;
    - CACI;

- SBT.

The 6-month estimate of tolerability of treatments (as measured by the ASEC), severity of depression (as measured by the MADRS), subjective quality of life (as measured by the EQ-5D), cognitive performance (as measured by the SBT), and comorbidity (as measured by the CACI) will be compared between the two groups of treatment through appropriate statistical methods (analysis of covariance with baseline value as an additional covariate or Mann-Whitney test (on changes) according to the variables distribution). Same outcomes will be studied using linear mixed models taking into account all assessments to evaluate the rate of change with shorter repeated evaluations and no need of missing imputation. Further, score changes in subscales will be evaluated in order to detect possible specific treatment-related side-effects.

A Cox proportional hazard model will be used to explore time to treatment withdrawal due to adverse events (secondary analysis). The proportional hazard assumption of the effects will be tested.

Adverse events will be tabulated. Nominal value for statistical significance will be set at 0.05, two tailed. A specific Statistical Analysis Protocol will be produced before the inclusion of the last patient. All analyses will be performed using STATA, release 13.1 or higher (StataCorp, College Station, TX, USA).

Considerations about pragmatism. The design of this study aims at achieving a high level of pragmatism. This approach will allow to minimize the risk of selection bias (particularly relevant when assessing fragile populations such as the elderly), to maintain standard routine clinical procedures as much as possible, and therefore to maximize the external validity and generalizability of results (Ford and Norrie, 2016). In synthesis, the following features will be employed to maximize pragmatism:

a. Patients will be enrolled on the basis of clinical judgment. No external operationalized diagnostic criteria of depressive episode will be applied;
b. No limitation to the setting, co-existing medical conditions (with the only exception of dementia) and pharmacotherapies will be applied;
c. A web-based application will be employed in order to simplify the process of recruitment, randomization, and collection of socio-demographic and clinical data. This will minimize the time deducted from ordinary clinical practice;
d. The employment of a hard, pragmatic primary outcome (withdrawal rate due to adverse events), along with the employment of blinded outcome assessors and statisticians, will allow to maintain an open-label design (much simpler for patients and staff), minimizing at the same time the risk of performance and detection bias;
e. The comparison group will be the SSRI class, and not one individual drug, which could introduce some degree of selection bias (i.e. excluding patients who did not benefit from a specific drug in the past);
f. A flexible dosing schedule will be employed, according to clinical judgment, within the recommended therapeutic range;
g. In order to avoid an unbalanced randomization, stratification according to the recruiting centre will be applied;
h. With the exception of the administration of three rating scales by a blinded outcome assessor, no further therapeutic or diagnostic interventions will be employed in addition to those usually performed in routine practice.

Trial Management

Coordinating Centre. The University of Verona will have the role of study coordinator and Prof. Corrado Barbui will be the principal investigator of the study. The Unit of Psychiatry of the AOUI Verona and the WHO Collaborating Centre for Research and Training in Mental Health and Service Evaluation of Verona have a longstanding experience in planning

and coordinating both experimental and observational research involving multiple centers across Italy (Barbui et al., 2011; Girlanda et al., 2014; Nosé et al., 2016). In particular, the coordinating center will:

- maintain constant contacts with local researchers of each center involved, in order to help them producing the documentation for the local Ethics Committee before the study starts;
- manage economical resources thanks to an administration expert;
- provide participating centers with basic training for the procedures of patients' enrollment and follow-up, and provide constant support throughout the entire study;
- store all relevant administrative documentation from each participating centers;
- coordinate the publication and dissemination planning.

Standard Operating Procedures. Standard Operating Procedures (SOPs) describing all phases of the study will be developed following a predefined template, consecutively numbered and circulated across sites to be commented and harmonized. Throughout the whole duration of the study, hard and electronic copies of the SOPs final and approved versions will be stored in dedicated archives.

Feasibility. All collaborating centres have previous experience in the field of clinical research, and many of them took part to previous multicentre projects coordinated by the Verona group (Barbui et al., 2011; Girlanda et al., 2014; Nosé et al., 2016; Carrà et al., 2016; Barbui et al., 2016). This study is characterized by many feasibility elements, including the following:

- the high frequency of depression in the elderly;
- the highly pragmatic approach;
- the employment of an electronic application which largely simplifies the procedures of randomization and data collection;
- the high number of recruiting centres, each with previous expertise in experimental research;
- the presence of a highly qualified collaborating unit (Mario Negri Institute, Milan) specifically dedicated to data management and statistical analysis.

Timing

	T0<br>Enrolment phase<br>(duration: 12 months)	T1<br>(1 month)	T2<br>(3 months)	T3<br>(6 months)
Review of criteria for inclusion in the study	X			
Informed consent document signed	X			
Randomization (allocation to treatment and number assigned)	X			
Recruitment Form	X			
ASEC		X	X	X
MADRS	X	X	X	X
EQ-5D	X	X	X	X
CACI	X	X	X	X
SBT	X	X	X	X
Follow-up form		X	X	X
Severe Adverse Event (SAE) Form		← ← any time → →		

First, the protocol should be approved by the EC of the Coordinating Center, which can take up to 3 months. Afterwards, the other centers will submit the protocol to the local ECs. Once this is formally approved, the center will begin the 12-months enrollment phase. On the basis of previous multicenter research experiences, we expect local ECs to finalize the study approval within a time span of about 6 months from the first to the last recruiting center. Each patient will be followed-up for 6 months, and assessed after 1, 3 and 6 months (see the attached schedule). Therefore, we expect no less than 27 months from the beginning of recruitment in the first center to the last follow-up in the last recruiting center. Once the follow-up of the last recruited patient is completed, a time period of 6 months is needed in order to complete the data entry, check the database quality, and perform statistical analysis. Another 3 months are needed in order to write the final study report. Therefore, the study will last 36 months from the submission of the protocol to the local EC to the production of the final report of study results.

Dissemination. The project will develop a strategy for optimal dissemination and uptake of its results, having in mind as main target groups the European Medicines Agency, the Italian AIFA, national and regional policy makers and planners, and the scientific community. Once the final report is available, the study results will be extensively disseminated to the international scientific community in the form of peer-reviewed journal articles, giving preference to open-access journals (fees are included in the budget). Also, abstracts and oral presentations in national and international meetings and conferences will represent an additional source of dissemination.

Good clinical practices and ethical aspects. This study will be conducted according to globally accepted standards of good clinical practice, as defined in the ICH E6 Guideline for Good Clinical Practice, 1 May 1996, in agreement with the Declaration of Helsinki and in keeping with local regulations.

The principal investigator and other collaborating investigators at the coordinating centre will ensure that all personnel involved in the trial will be adequately qualified, informed about the protocol, the study treatments, their trial-related duties and functions, and about any amendments of procedures as described in the protocol. The coordinating centre will maintain a list of local investigators and other appropriately qualified persons involved in the study.

Before being enrolled in the study, subjects will consent to participate after the nature, scope, and possible consequences of the clinical trial have been explained in a form understandable to them. An informed consent document that includes

both information about the study and the consent form will be given to participants. This document will contain all the elements required by the ICH E6 Guideline for Good Clinical Practice and any additional element required by local regulations. The document will be in a language understandable to the participants and will specify who informed the subject. The person who informs the subject will be a physician. After reading the informed consent document, the subject, or his/her legal representative, must give consent in writing. The subject's consent must be confirmed at the time of consent by the personally dated signature of the subject and by the personally dated signature of the person conducting the informed consent discussion.

According to the ICH E6 Guideline for Good Clinical Practice, subjects who will be enrolled in the trial with the consent of the subjects' legally acceptable representative will be informed about the trial to the extent compatible with the subjects' understanding and, if capable, the subject will be asked to sign and personally date the written informed consent.

The Promoter and the participating centers will process the personal data of the patients, each for the areas of their competence and in accordance with the responsibilities established by the rules of good clinical practice (Legislative Decree 211/2003) and in compliance with the laws and regulations in force in data protection law including the European Data Protection Regulation 2016/679.

The highly pragmatic design will minimize any potential risk related to the complexity of the study design. In line with this, an assessment phase before recruitment will not be needed, and procedures of treatment allocation and main data collection are designed in order to minimize the time deduction to ordinary clinical practice. Afterwards, patients will be asked to provide further data through the administration of rating scales. Patients will be prescribed with medications already available in the market, and approved for treating depression. Therefore, risks for the patient will be limited to those already known from previous studies.

In accordance with the Declaration of Helsinki, the patients' confidentiality will be preserved at all times and the contents of the recruitment and follow-up forms will not be disclosed to any third party. The data collected in the study corresponding to a patient will be recorded anonymously. Patients will be identified by a unique identifying number, both in the RF and FUF forms and in the database. Total confidentiality of data is guaranteed, especially the identity of participants. The local coordinator of each centre will have the duty to produce a password-protected file, accessible only to him/her, reporting all unique identifiers and the corresponding name of the participant.

The steering committee of the study will supervise all the ethical issues related to the trial. The EAB will help the consortium keep high ethical standards, which will ultimately enhance the quality of research, increase its likely social impact, promote integrity and a better alignment of VESPA with social needs and expectations. The EAB will make sure that relevant steps are undertaken to minimize risks or provide solutions in case relevant adverse events are detected during screening or during the course of the study. In synthesis, the EAB will indirectly supervise three main aspects: (1) recruitment, inclusion and exclusion criteria, and informed consent procedures; (2) data management (protection and privacy); (3) vulnerability of the population.

Study investigators will ensure that the trial is adequately monitored. The purpose of trial monitoring will be to verify that the reported data are accurate, complete, and verifiable from source documents. Recruiting centres will allow the coordinating centre, at its discretion, to monitor and audit the conduct of any procedure related to the study. This includes the right to inspect any facility being used for the study and to examine any relevant procedures and records. In particular, the principal investigator will visit each study centre at least one time during the phase of enrolment or follow-up.

Expected impact

Results from this study can have a profound impact on everyday clinical practice, considering (a) the overall psychological, medical and economic burden of depression in the elderly, and (b) the few available pharmacological

alternatives for treating these patients. If the hypothesis of a better tolerability and safety of vortioxetine will be confirmed, this drug could become a reference first- or second-line drug for the treatment of depression in the elderly. A new safe and effective treatment for such a vulnerable population has the potential of increasing the number of treatment responders, ameliorating psychological well-being and quality of life of elder patients, reducing at the same time health care costs related to adverse events (including severe conditions such as falls, fractures, bleeding, hyponatraemia, QTc alterations at risk for arrhythmias), hospitalizations, and poor outcomes of medical comorbidities. Considering the pragmatic nature of the study, we expect that the study results will be immediately extended to Italian doctors and patients who will require no training or specific information exchange activities to modify their clinical practice: if vortioxetine is more tolerated than the SSRIs, clinical practice will be modified accordingly, and vortioxetine will be a first-line treatment in this patient group; if vortioxetine is not more tolerated than the SSRIs, vortioxetine, instead of being used without any guidance, as currently happens in ordinary Italian practice, will have a clearer place in the treatment of elderly depressed patients, being for example reserved as a second-line treatment when SSRIs are not effective. This study will additionally have regulatory implications, considering that the EMA states as follows "Caution is advised when treating patients ≥ 65 years of age with doses higher than 10 mg vortioxetine once daily for which data are limited" (EMA, 2014b). We expect that this statement will be reformulated in view of the study results: if vortioxetine is more tolerated than the SSRIs, by mentioning its favorable tolerability profile; if vortioxetine is less well tolerated than the SSRIs, by further reinforcing the cautionary statement.

Insurance

The promoter stipulated an insurance policy covering all patients included in the study.

Conflict of interest disclosure

The authors have no competing interests to disclose.

Study flow-chart

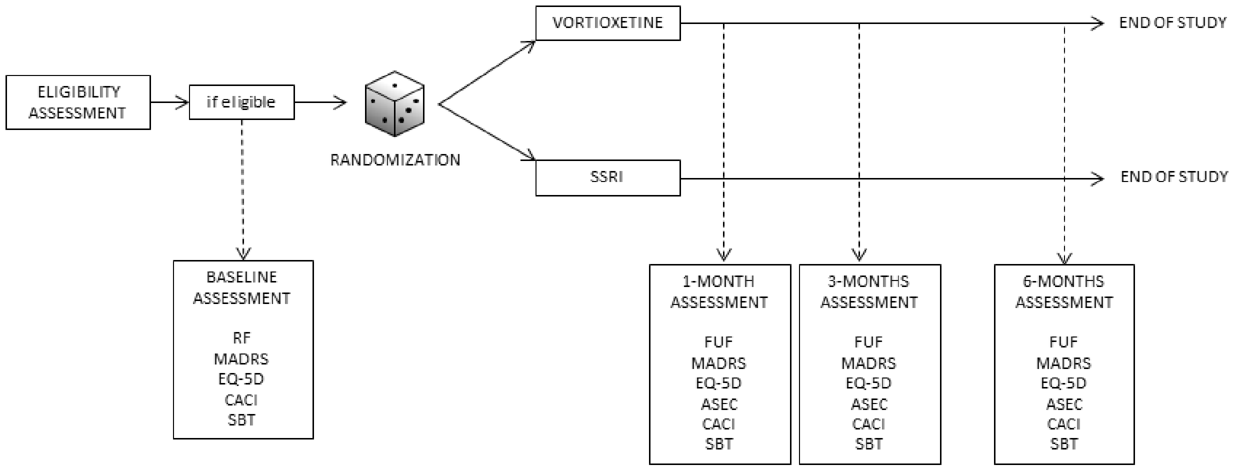

Legend. RF=recruitment form; FUF=follow-up form; MADRS=Montgomery–Åsberg Depression Rating Scale; UKU=Udvalg for Kliniske Undersogelser Side Effect Rating Scale: EQ-5D=EuroQual 5 Dimensions

References

Barbui C, Furukawa TA, Cipriani A. Effectiveness of paroxetine in the treatment of acute major depression in adults: a systematic re-examination of published and unpublished data from randomized trials. CMAJ 2008,178 (3):296–305

Barbui C, Accordini S, Nosè M, et al. Aripiprazole versus haloperidol in combination with clozapine for treatment-resistant schizophrenia in routine clinical care: a randomized, controlled trial. J Clin Psychopharmacol 2011,31(3):266-73

Carrà G, Crocamo C, Bartoli F, et al. First-generation antipsychotics and QTc: any role for mediating variables? Hum Psychopharmacol. 2016;31(4):313-8

Charlson M, Szatrowski TP, Peterson J, Gold J. Validation of a combined comorbidity index. J Clin Epidemiol. 1994;47(11):1245-51

Cipriani A, Furukawa TA, Salanti G, Chaimani A, Atkinson LZ, Ogawa Y, Leucht S, Ruhe HG, Turner EH, Higgins JPT, Egger M, Takeshima N, Hayasaka Y, Imai H, Shinohara K, Tajika A, Ioannidis JPA, Geddes JR. Comparative efficacy and acceptability of 21 antidepressant drugs for the acute treatment of adults with major depressive disorder: a systematic review and network meta-analysis. Lancet. 2018. pii: S0140-6736(17)32802-7

Coupland C, Dhiman P, Morriss R, et al. Antidepressant use and risk of adverse outcomes in older people: population based cohort study. BMJ, 2011;343:d4551

Du Jardin KG, Liebenberg N, Muller HK, et al. Differential interaction with the serotonin system by S-ketamine, vortioxetine, and fluoxetine in a genetic rat model of depression. Psychopharmacology 2016,233(14):2813-25

European Medicines Agency (EMA). Brintellix EPAR - Public assessment report 2014a. http://www.ema.europa.eu/docs/en_GB/document_library/EPAR_-_Public_assessment_report/human/002717/WC500159447.pdf

European Medicines Agency (EMA). Annex I. Summary Of Product Characteristics. 2014b. Available: http://www.ema.europa.eu/docs/en_GB/document_library/EPAR_-_Product_Information/human/002717/WC500159449.pdf

EuroQol Group. EuroQol-a new facility for the measurement of health-related quality of life. Health Policy 1990,16(3):199-208.

GBD 2015 Risk Factors Collaborators. Global, regional, and national comparative risk assessment of 79 behavioural, environmental and occupational, and metabolic risks or clusters of risks, 1990-2015: a systematic analysis for the Global Burden of Disease Study 2015. Lancet. 2016,388(10053):1659-1724

Girlanda F, Cipriani A, Agrimi E, et al. Effectiveness of lithium in subjects with treatment-resistant depression and suicide risk: results and lessons of an underpowered randomised clinical trial. BMC Res Notes 2014, 7:731

Heisel MJ, Grek A, Moore SL, et al. National guidelines for seniors' mental health: The assessment of suicide risk and prevention of suicide. Can J Geriatr 2006;9(suppl2):S65-S70

Katona C, Hansen T, Olsen CK (2012). A randomized, double-blind, placebo-controlled, duloxetine-referenced, fixed-dose study comparing the efficacy and safety of Lu AA21004 in elderly patients with major depressive disorder. Int Clin Psychopharmacol 27: 215–23

Katzman R, Brown T, Fuld P. Validation of a short orientation-memory-concentration test of cognitive impairment. Am J Psychiatry. 1983;140:734–9

Kelliny M, Croarkin PE, Moore KM, Bobo WV. Profile of vortioxetine in the treatment of major depressive disorder: an overview of the primary and secondary literature. Ther Clin Risk Manag. 2015,11:1193-212

Koesters M, Ostuzzi G, Guaiana G, Breilmann J, Barbui C. Vortioxetine for depression in adults. Cochrane Database of Systematic Reviews 2015 , Issue 2 . Art. No.: CD011520

Mahableshwarkar AR, Zajecka J, Jacobson W, et al. A Randomized, Placebo-Controlled, Active-Reference, Double-Blind, Flexible-Dose Study of the Efficacy of Vortioxetine on Cognitive Function in Major Depressive Disorder. Neuropsychopharmacology. 2015,40(8):2025-37

McIntyre RS, Lophaven S, Olsen CK. A randomized, double-blind, placebo-controlled study of vortioxetine on cognitive function in depressed adults. Int J Neuropsychopharmacol. 2014,17:1557–67

Mottram PG, Wilson K, Strobl JJ. Antidepressants for depressed elderly. Cochrane Database Syst Rev 2006; CD003491

NICE. Depression in adults: recognition and management. Updated 2016. https://www.nice.org.uk/guidance/cg90

Nosè M, Bighelli I, Castellazzi M, et al. Prevalence and correlates of QTc prolongation in Italian psychiatric care: cross-sectional multicentre study. Epidemiol Psychiatr Sci. 2016, 25(6):532-40

Pocock SJ. Clinical Trials: A Practical Approach. Wiley; 1983

Sanchez C, Asin KE, Artigas F. Vortioxetine, a novel antidepressant with multimodal activity: review of preclinical and clinical data. Pharmacol Ther 2015, 145C: 43–57

Steenland K, Karnes C, Seals R, et al. Late-life depression as a risk factor for mild cognitive impairment or Alzheimer's disease in 30 US Alzheimer's disease centers. J. Alzheimers Dis. 2012, 31:265–75

Uher R, Farmer A, Henigsberg N, et al. Adverse reactions to antidepressants. Br J Psychiatry 2009;195: 202- 210

US Food and Drug Administration (FDA). Drug Approval Package. Brintellix (vortioxetine) Tablets. 2013. http://www.accessdata.fda.gov/drugsatfda_docs/nda/2013/204447Orig1s000TOC.cfm

WHO. mhGAP. 2012. http://www.who.int/mental_health/mhgap/evidence/depression/q2/en/

Supplement B. Participant information sheet (version 01; 06/04/2018)

Documento informativo per il partecipante relativo alla sperimentazione clinica:

Valutare la sicurezza e l'efficacia di vortioxetina in confronto ai farmaci antidepressivi SSRI in pazienti anziani con depressione: uno studio pragmatico, multicentrico, in aperto, a gruppi paralleli, di superiorità, randomizzato.

VESPA (Vortioxetine in the Elderly vs. SSRIs: a Pragmatic Assessment)

Gentile Signora/Egregio Signore,

____________________ ____________________

(cognome) *(nome)*

Le è stato chiesto di partecipare ad uno studio clinico randomizzato che si propone di testare il farmaco antidepressivo vortioxetina per quanto riguarda la riduzione degli effetti collaterali e il miglioramento dei sintomi depressivi rispetto ai farmaci antidepressivi della categoria SSRI (inibitori selettivi della ricaptazione della serotonina), che includono sertralina, citalopram, escitalopram, paroxetina, fluoxetina, fluvoxamina. Prima che Lei prenda una decisione in merito alla partecipazione, è importante che comprenda il motivo dello studio e cosa Le sarà chiesto di fare, qualora decidesse di prendevi parte. Lo sperimentatore responsabile ed i suoi collaboratori, oltre alle spiegazioni che Le forniranno durante questo colloquio, sono a Sua completa disposizione per qualsiasi chiarimento. Questo progetto di ricerca, finanziato dall'Agenzia Italiana del Farmaco all'interno del Bando 2016 per la Ricerca Indipendente, è coordinato dall'Università di Verona e verrà condotto in altri 13 centri italiani.

Questo documento ha lo scopo di fornirle un'informazione corretta e completa affinché Lei possa esprimere una scelta libera e consapevole.

Sperimentatore principale

Prof. Corrado Barbui

Centro OMS di ricerca sulla Salute Mentale, Dipartimento di Neuroscienze, Biomedicina e Movimento, Università di Verona

Telefono: 045 8126418; 045 8124884

A cosa serve questo documento?

Questo documento ha lo scopo di fornirle un'informazione corretta e completa sulle caratteristiche dello studio clinico a cui è stato invitato a partecipare. Lo sperimentatore principale ed i suoi collaboratori, oltre alle spiegazioni che Le forniranno durante questo colloquio, sono a Sua completa disposizione per qualsiasi chiarimento.

Perché mi è stato chiesto di partecipare a questo studio?

Lei fa parte della popolazione che verrà considerata in questo studio, ovvero pazienti anziani (età uguale o maggiore di 65 anni), affetti da un attuale episodio di depressione maggiore che richiede un trattamento farmacologico con antidepressivi. Lo sperimentatore ritiene che possieda i requisiti necessari per entrare nello studio. Le è stato chiesto di considerare la possibilità di aderirvi, per valutare se il nuovo farmaco vortioxetina può contribuire a migliorare il Suo stato di salute psicologica e a ridurre il rischio di effetti collaterali rispetto ai farmaci antidepressivi della categoria SSRI (inibitori selettivi delle ricaptazione della serotonina). Questo studio sperimentale sarà condotto in 14 centri italiani e vi parteciperanno circa 360 persone.

Qual è lo scopo dello studio?

L'obiettivo di questo studio clinico è quello di esaminare se il nuovo farmaco vortioxetina ha un minor rischio di effetti collaterali rispetto ai farmaci antidepressivi della categoria SSRI (inibitori selettivi delle ricaptazione della serotonina, che sono attualmente l'intervento standard più comunemente utilizzato), ed è efficace nel ridurre i sintomi depressivi e nel migliorare la qualità della vita.

Qual è l'intervento che viene testato?

Il farmaco che viene testato in questo studio si chiama vortioxetina, è un farmaco antidepressivo. L'intervento è stato già sperimentato in precedenti studi clinici randomizzati.

Sono obbligato a partecipare?

No. La decisione di partecipare allo studio dipende solo da Lei. E' completamente volontaria. Se preferisce non partecipare non deve fornire spiegazioni. Riceverà comunque tutte le indagini e gli eventuali interventi attualmente disponibili per la Sua condizione medica e psicologica.

Cosa accadrà se decido di partecipare allo studio?

Se desidera prendere in considerazione la possibilità di parteciparvi, Le sarà consegnata questa scheda informativa, da leggere e conservare. Avrà la possibilità di chiedere tutte le spiegazioni che desidera a riguardo. Le sarà chiesto di firmare il modulo di consenso, in allegato. Solo dopo che Lei avrà firmato la dichiarazione di consenso, verrà assegnato a uno dei due gruppi di trattamento in studio.

Riceverò vortioxetina o il trattamento standard (antidepressivi SSRI)?

Lei avrà il 50% di probabilità di ricevere vortioxetina. Se non riceverà vortioxetina, riceverà un farmaco antidepressivo SSRIs, ovvero uno tra sertralina, citalopram, escitalopram, paroxetina, fluoxetina, fluvoxamina, in base al giudizio del medico. Tali farmaci sono considerati attualmente l'intervento standard previsto nella normale pratica clinica.

Chi decide se riceverò vortioxetina o il trattamento standard (antidepressivi SSRI)?

Qualora decidesse di partecipare allo studio, lei sarà assegnato in modo casuale a ricevere l'intervento sperimentale (vortioxetina) o l'intervento standard (un farmaco della categoria SSRI, a discrezione del medico), in base ad un sistema computerizzato che non può essere condizionato né dal medico, né dal partecipante.

Sarò a conoscenza del trattamento che riceverò?

Sì, lo studio sarà condotto "in aperto". Questo significa che lei saprà quale trattamento riceverà.

Quanto dura lo studio?

Lo studio dura 6 mesi, durante i quali è prevista la partecipazione a tre colloqui di valutazione medica e psicologica in aggiunta al colloquio odierno, in linea a quanto previsto nella normale pratica clinica.

Quali tipi di esame o di procedure sono previste in questo studio?

Le visite di controllo verranno effettuate con la stessa frequenza prevista dalla normale pratica clinica. Nel corso di tali visite, oltre al consueto monitoraggio clinico e terapeutico svolto dal medico psichiatra, verranno compilati dei questionari clinici e psicologici per valutare sistematicamente la presenza di eventuali effetti collaterali, l'intensità della sintomatologia depressiva, la qualità della vita, e l'eventuale presenza di condizione mediche rilevanti. La compilazione di tali questionari richiederà all'incirca 10-15 minuti.

Lo studio è stato approvato da un Comitato Etico?

Sì, lo studio è stato approvato dal Comitato Etico per la sperimentazione clinica delle provincie di Verona e Rovigo.

Cosa devo fare se decido di non aderire o di ritirarmi?

Lei può decidere di ritirarsi da questo studio in qualsiasi momento, comunicandolo allo sperimentatore e senza dover fornire alcuna spiegazione.

Cosa devo fare se decido di partecipare? Quale sarà il mio impegno?

All'ingresso dello studio, lo sperimentatore verificherà che Lei soddisfi tutti i criteri previsti per la partecipazione. Qualora acconsenta a partecipare allo studio, il medico raccoglierà alcune informazioni sul suo stato psicologico, sulla sua storia personale e clinica, e le verranno somministrati dei questionari clinici per misurare eventuali effetti collaterali, i sintomi depressivi, la qualità della vita, e la presenza di eventuali malattie. Conclusa questa raccolta di informazioni (che richiederà indicativamente 10-15 minuti) potrà essere assegnato a uno dei due trattamenti in studio. Se sarà assegnato alla vortioxetina, questa le potrà essere prescritta in formulazione orale (gocce o compresse, a discrezione del medico prescrittore, sulla base della normale valutazione e pratica clinica), ad un dosaggio iniziale pari a 5 mg (milligrammi) al giorno, che potrà essere gradualmente aumentato nel corso dello studio, in caso di risposta insoddisfacente, fino ad un massimo di 20 mg al giorno. Il medico prescrittore, sulla base della normale valutazione e pratica clinica, potrà indicarle di assumere tale farmaco in una o più somministrazioni giornaliere. Anche qualora fosse assegnato al gruppo dei farmaci SSRI, la formulazione (compresse o gocce) e la posologia (dosaggio, numero di somministrazioni giornaliere, ed eventuale graduale aumento del dosaggio) del farmaco verranno decise sulla base della normale valutazione e pratica clinica. I farmaci SSRI possono essere prescritti nell'anziano ai seguenti dosaggi: (a) sertralina, da 50 a 200 mg al giorno; (b) paroxetina, da 20 a 40 mg al giorno; (c) citalopram, da 10 a 20 mg al giorno; (d) escitalopram, da 5 a 10 mg al giorno; (e) fluoxetina, da 20 a 60 mg al giorno; (f) fluvoxamina, da 100 a 300 mg al giorno.

Successivamente le sarà chiesto di partecipare ad altri tre colloqui di valutazione: uno dopo un mese dall'inizio dello studio, uno dopo tre mesi, e uno dopo sei mesi. Le date dei colloqui di valutazione clinica saranno stabilite all'inizio dello studio e le verrà consegnato un promemoria cartaceo. Inoltre, lo sperimentatore o un suo collaboratore La contatterà una settimana prima ed un giorno prima di ogni colloquio di valutazione clinica per confermare la visita programmata.

È molto importante che Lei partecipi ai colloqui di valutazione clinica programmati. Inoltre, Le sarà chiesto di informare lo sperimentatore di eventuali problemi/disturbi che potranno insorgere e di ogni variazione riguardante la Sua salute.

Quali benefici potrò attendermi dalla partecipazione allo studio?

Il beneficio atteso è quello di ottenere un miglioramento della Sua condizione depressiva partecipando allo studio, riducendo nello stesso tempo il rischio di effetti collaterali rispetto ai trattamenti standard. Tuttavia, tale beneficio non può esserle garantito.

Quali potrebbero essere i rischi associati alla partecipazione allo studio?

I rischi associati alla partecipazione a questo studio potrebbero essere associati al non miglioramento della condizione depressiva di cui lei soffre, nonché a possibile effetti collaterali che caratterizzano sia l'intervento (vortioxetina), sia il trattamento standard (SSRI). In particolare, gli effetti collaterali comuni di **vortioxetina** (che hanno una frequenza che va da 1 paziente su 100 a 1 paziente su 10) includono sogni anormali, capogiro, diarrea, costipazione, vomito, prurito, mentre la nausea è molto comune (frequenza superiore a 1 paziente su 10). I farmaci **SSRI** possono avere profili leggermente differenti, ma in generale gli effetti collaterali comuni (che hanno una frequenza che va da 1 paziente su 100 a 1 paziente su 10) includono faringite, diminuzione o aumento dell'appetito, depersonalizzazione, incubi, ansia, agitazione, nervosismo, riduzione della libido, bruxismo, parestesia, tremori, ipertonia, disgeusia, disturbi dell'attenzione, disturbi della vista, tinnito, palpitazioni, vampate di calore, sbadigli, dolore addominale, vomito, stipsi, dispepsia, flatulenza, eruzione cutanea, iperidrosi, artralgia, mialgia, disfunzione erettile, dolore al torace, malessere. Gli effetti molto comuni (frequenza superiore a 1 paziente su 10) includono affaticamento, mancata eiaculazione, diarrea, nausea, bocca secca, capogiri, sonnolenza, cefalea, insonnia.

Potrò cambiare idea dopo aver accettato di partecipare?

Si. Lei potrà decidere di ritirare il consenso e interrompere il trattamento, in qualsiasi momento, anche a studio avviato, senza dover fornire giustificazioni a meno che la decisione non derivi dalla comparsa di disturbi o effetti indesiderati o non previsti, nel qual caso dovrà fornire allo sperimentatore tutte le informazioni del caso. La Sua decisione non avrà ripercussioni sull'assistenza e sulle cure che dovrà ricevere in futuro.

Il mio medico di fiducia sarà informato?

Previa sua autorizzazione, il Suo medico di fiducia sarà informato con apposita lettera della Sua partecipazione e potrà anche contattare il responsabile dello studio per qualsiasi informazione.

Il trattamento sperimentale potrebbe essere interrotto o sospeso?

Si. Lo sperimentatore potrebbe interrompere lo studio in qualsiasi momento, anche contro la Sua volontà, qualora lo ritenesse necessario per la Sua salute, o per la corretta conduzione della ricerca.

Cosa accadrà se subentrassero problemi: infortunio o danni correlati allo studio?

Qualora dovessero verificarsi effetti collaterali, indesiderati, o danni alla Sua salute, riconducibili allo studio, Lei dovrà informare tempestivamente il medico sperimentatore che Le fornirà le relative informazioni. Il Centro OMS di ricerca sulla Salute Mentale (Università di Verona) che ha commissionato lo studio, ha stipulato per questo una polizza assicurativa con la compagnia Lloyd's.

Quanto mi costerà partecipare allo studio?

La Sua partecipazione allo studio di ricerca non comporterà per Lei alcun aggravio di spesa. Tutti i costi associati alla prescrizione e somministrazione dei farmaci in studio, così come ai colloqui e alla somministrazione dei test di valutazione saranno coperti dall'Università di Verona e dal Sistema Sanitario Nazionale, come nella normale pratica clinica.

Chi organizza e finanzia lo studio di ricerca?

Lo studio è organizzato e coordinato dal Centro OMS di ricerca sulla Salute Mentale, Dipartimento di Neuroscienze, Biomedicina e Movimento, Università di Verona. L'Università di Verona riceve un finanziamento dall'AIFA (Agenzia Italiana del Farmaco) per lo svolgimento di questo studio.

La mia partecipazione resterà riservata? Come saranno usati i miei dati personali?

Le informazioni che la riguardano personalmente saranno sempre trattate come confidenziali secondo quanto previsto dalle normative vigenti (Decreto Legislativo n. 196 del 30 Giugno 2003, "Codice in materia di protezione dei dati personali"). I suoi dati personali quali le generalità e le altre informazioni anagrafiche, nonché i dati idonei a rivelare il suo stato di salute saranno gestiti ed archiviati esclusivamente per finalità connesse agli obiettivi dello studio. La gestione e l'informazione dei dati sarà gestita in forma cartacea e informatizzata. Nel caso in cui i risultati di questo studio fossero oggetto di pubblicazioni scientifiche, la sua identità rimarrà riservata.

Chi posso contattare per ulteriori informazioni, necessità, richieste d'aiuto?

Se desiderasse ricevere ulteriori informazioni su questo studio può contattare:

Prof. Corrado Barbui

Centro OMS di ricerca sulla Salute Mentale, Dipartimento di Neuroscienze, Biomedicina e Movimento, Università di Verona; Telefono: 045 8126418; 045 8124884

Dr. Giovanni Ostuzzi

Centro OMS di ricerca sulla Salute Mentale, Dipartimento di Neuroscienze, Biomedicina e Movimento, Università di Verona; Telefono: 045 8124063

DICHIARAZIONE DI CHI INFORMA

Io sottoscritta/o ______________________________________ dichiaro di aver informato il/la paziente e discusso dello scopo e della natura dello studio clinico in oggetto, di aver risposto ad ogni sua domanda riguardo la natura, l'impegno, le procedure, i rischi e i benefici della partecipazione al presente studio di ricerca.

Dal colloquio sono emersi elementi sufficienti, per affermare che il paziente ha compreso natura, scopo e quant'altro gli/le viene chiesto conseguentemente alla Sua partecipazione

Verona, __________ Firma del professionista ___________________________

FIRMA INFORMATIVA

Io sottoscritta/o ________________________________ dichiaro di aver ricevuto e discusso tutte le informazioni che mi hanno permesso di comprendere lo scopo, la natura, i rischi e i benefici dello studio clinico cui mi si chiede di partecipare

Verona, __________ Firma del partecipante ______________________________

Verona, __________ Firma del testimone ________________________________

Supplement C. Informed consent form (version 01; 06/04/2018)

ESPRESSIONE DEL CONSENSO – SPERIMENTAZIONE CLINICA

Luogo e data ____

Io sottoscritto/a (NOME E COGNOME) ____________________________ *nato il* ______________________

dichiaro

di accettare la proposta di sottopormi alla sperimentazione clinica

Valutare la sicurezza e l'efficacia di vortioxetina in confronto ai farmaci antidepressivi SSRI in pazienti anziani con depressione: uno studio pragmatico, multicentrico, in aperto, a gruppi paralleli, di superiorità, randomizzato.

VESPA (Vortioxetine in the Elderly vs. SSRIs: a Pragmatic Assessment)

Sono stato/a informato/a in modo chiaro e completo, ricevendo la relativa informativa, circa gli scopi dello studio e le metodiche dello stesso, in particolare sono consapevole della necessità di osservare le indicazioni e le regole che mi sono state illustrate e che ho compreso.

Sono a conoscenza dei benefici che mi possono derivare dalla partecipazione allo studio, ma anche degli eventuali rischi e di tutti i disagi connessi.

Mi è stato spiegato che dal nuovo trattamento potrebbero attendersi dei miglioramenti rispetto agli approcci terapeutici ad oggi in uso.

Ad ogni modo mi è stato assicurato che non subirò, prevedibilmente, alcun aggravamento delle mie condizioni cliniche né vi sarà un ritardo nei tempi solitamente necessari, in casi analoghi, per il controllo del mio stato psicologico.

Sono consapevole che in qualsiasi momento potrò sospendere la sperimentazione ed esigere di essere curato/a con il trattamento standard per la mia condizione psicologica, senza obbligo da parte mia di motivare la decisione, a meno che la stessa non derivi dalla comparsa di disturbi o effetti indesiderati o non previsti, nel qual caso mi impegno sin da ora a comunicarne tempestivamente al medico sperimentatore natura ed entità.

Dichiaro che il mio consenso è espressione di una libera decisione, non influenzata da promesse di denaro o di altri benefici, né da obblighi di gratitudine o di amicizia e/o parentela nei confronti del medico sperimentatore.

□ *acconsento*

□ *non acconsento*

Autorizzo sin d'ora l'utilizzo e la divulgazione, in forma anonima e per sole finalità scientifiche e amministrative e nell'osservanza delle vigenti norme sulla tutela della riservatezza, dei risultati della sperimentazione, compresi i dati clinici che mi riguardano.

Verona, ____________ *Firma del partecipante*________________________________

Verona, ____________ *Firma del Testimone* ________________________ *(se appropriato)*

Verona, ____________ *Firma dello Sperimentatore* ____________________________

Supplement D. Approval by the Ethics Committee of the Coordinating Centre

AZIENDA OSPEDALIERA UNIVERSITARIA INTEGRATA
VERONA

(D.Lgs. n. 517/1999 - Art. 3 L.R.Veneto n. 18/2009)

DIPARTIMENTO DIREZIONE MEDICA OSPEDALIERA

COMITATO ETICO PER LA SPERIMENTAZIONE CLINICA
DELLE PROVINCE DI VERONA E ROVIGO

UFFICIO DI SEGRETERIA TECNICO-SCIENTIFICA DEL COMITATO ETICO
c/o SERVIZIO DI FARMACIA
Borgo Trento - P.le A. Stefani, 1 - 37126 Verona - Tel. 045 8123236 - Fax 045 8123177
e-mail: comitatoetico.veronarovigo@aovr.veneto.it
PEC: comitatoetico.aovr@pecveneto.it

Prot n 61211 del 19/09/2018

Sperimentatore: Prof. Barbui Corrado - Psichiatria - (UOC) - Azienda Ospedaliera Universitaria Integrata - Verona

Direttore U.O.: Prof.ssa Ruggeri Mirella - Psichiatria - (UOC) - Azienda Ospedaliera Universitaria Integrata - Verona

Promotore: A.O. UNIVERSITARIA INTEGRATA DI VERONA

NRC: A.O. UNIVERSITARIA INTEGRATA DI VERONA

Oggetto: Prog. 1903CESC - Studio Clinico: Valutare la sicurezza e l'efficacia di vortioxetina in confronto ai farmaci antidepressivi SSRI in pazienti anziani con depressione: uno studio pragmatico, multicentrico, in aperto, a gruppi paralleli, di superiorità, randomizzato. - Codice Protocollo: VESPA - Codice Eudract: 2018-001444-66

In riferimento alla richiesta di autorizzazione dello studio in oggetto, si trasmettono le decisioni del Comitato Etico per la Sperimentazione Clinica delle Province di Verona e Rovigo riunitosi in data **12/09/2018.**

Si rammenta che per l'attivazione della sperimentazione è necessario attendere:

1. L'autorizzazione da parte dell'Autorità Competente AIFA
2. ove previsto, la ricezione dell'autorizzazione della propria Amministrazione

Si ricorda che:

- Lo Sperimentatore è tenuto a segnalare al Comitato Etico l'arruolamento del primo paziente.
- Al termine della Sperimentazione, lo Sperimentatore dovrà inviare al Comitato Etico la relazione finale, al termine di ogni anno inoltre lo Sperimentatore dovrà inviare altresì la relazione annuale

Verona, 12/09/2018

D'Ordine del Presidente del Comitato Etico
delle Province di Verona e Rovigo
L'Ufficio di Segreteria
Dott.ssa Ilaria Bolcato

Ilaria Bolcato

Supplement E. Deviations from the original protocol and rationale

We made the following deviations from the original protocol:

a. We added the outcome "ASEC highest score" in addition to the original outcome "ASEC mean score", as we considered that the mean score might not accurately reflect the severity of adverse events, as this might be the result of time periods when adverse events occurred (or were more severe) and time periods when adverse events did not occur (or were less severe) for various reasons, including treatment discontinuation. Therefore, the highest score on the ASEC probably is the best proxy of the overall severity of adverse events occurred during the study follow-up;

b. In addition to self-harm episodes and complete suicide episodes, we also assessed episodes of attempted suicide. We grouped these outcomes under the label "suicidal behaviours", and slightly changed the terminology according to current standards in this research field: non-suicidal self-injury, suicide attempt, and death by suicide;

c. Although in the original protocol we aimed to perform "a multivariable analysis through a Poisson regression model with a robust error variance in order to adjust for the potential confounding effect", we actually performed "a multivariable analysis through a generalized linear mixed model (GLMM) with centre as a random effect and adjusting for the potential confounding effect of prognostic factors", as GLMM is currently considered the standard approach in such cases (see White N & Barnett A. Infection Control & Hospital Epidemiology 2019: 40(8), 910-7);

d. In addition to the last observation carried forward (LOCF) approach, we additionally performed "a multiple imputation approach, using SAS' Procedure Missing Imputation (PROC MI) which imputes a plausible value for each missing data using a predictive mean matching method for numeric variables, and discriminant function method for categorical variables", which is currently considered the reference approach in such cases (see Jakobsen et al. BMC Med Res Methodol 2017: 17, 162);

e. For continuous outcomes MADRS, EQ-5D, and SBT, according to the pre-planned statistical analysis described in the protocol, we compared the mean scores at the end of follow-up employing an analysis of covariance with baseline value as an additional covariate, which can be considered a more accurate approach compared to simply comparing mean changes in the two arms. However, as the analysis of the mean change was also mentioned in the original protocol, we performed this analysis as well (see Table F1 below). Results for the outcomes MADRS and SBT are consistent with the original analyses, while for the EQ-5D this analysis shows a statistically significant advantage for SSRIs over vortioxetine. In the original analysis (main manuscript, Table 2), we found such trend although it was not statistically significant (N=356; Estimate -3.49; 95% CI -7.59 to 0.61; p=0.0954). Additional analyses (scores at 1 and 3 months, and multiple imputation analysis - main manuscript, Table 2) are all consistent towards a lack of statistical significance of such differences between the two arms;

f. For the analysis of the SBT score, we removed participants who were diagnosed with dementia throughout follow-up;

g. In addition to two-tailed p-values, we performed also one-tailed tests, as shown in the Table F2 below;

h. In addition to the main analysis on SBT mean scores according to the ITT principle (see Table 2, main text), we re-analysed data removing those individuals who were diagnosed with dementia throughout the study (see Table F3 below).

Table E1. Comparing mean change of scores from baseline to month 6 in the two arms using a Wilcoxon Rank-Sum Test for two samples (or Mann-Whitney test). Analyses were performed on the ITT population using a LOCF approach to impute missing values.

Rating scale	Treatment	N	Mean change from baseline to month 6	95% CI inferior	95% CI superior	Standard deviation	p-value
MADRS	Vortioxetine	179	-12·73	-14·24	-11·22	10·22	0·13
	SSRI	178	-14·49	-16·12	-12·87	10·97	
EQ-5D	Vortioxetine	178	10·08	6·51	13·65	24·11	*0·019*
	SSRI	178	16·57	12·70	20·44	26·15	
SBT	Vortioxetine	179	0·14	-0·53	0·81	4·53	*0·0056*
	SSRI	177	-1·07	-1·79	-0·34	4·90	

Table E2. Analysis of the Short Blessed Test (SBT) after removing at each timepoint individuals with a diagnosis of dementia according to the Charlson Age Comorbidity Index (CACI).

	**Vortioxetine**	**SSRIs**	**Without adjustment**			**With adjustment**		
**Continuous outcomes**	**mean (SD)**	**mean (SD)**	**N**	**Estimate (95% CI)**	**p-value**	**N**	**Estimate (95% CI)**	**p-value**
**SBT**								
Baseline	4·41 (5·05)	4·63 (5·16)		-	-		-	-
Score 1 month (LOCF)	4·37 (5·31)	4·80 (5·81)	356	-0·28 (-1·15 to 0·59)	0·53	336	-0·06 (-0·99 to 0·88)	0·91
Score 3 months (LOCF)	4·10 (5·13)	3·93 (5·21)	354	0·31 (-0·54 to 1·15)	0·48	335	0·19 (-0·71 to 1·09)	0·68
Score 6 months (LOCF)	4·45 (5·95)	3·57 (5·11)	353	1·06 (0·14 to 1·98)	0·025	334	1·12 (0·14 to 2·10)	0·025
Score 6 months (multiple imputation)	-	-	-	0·93 (-0·11 to 1·98)	0·081	-	0·81 (-0·29 to 1·91)	0·15

Supplement F. Assessment of pragmatism with the tool PRECIS-2

Table F1. Scoring of PRECIS-2 tool. PRECIS 5-point Likert Scale score: (1) Very Explanatory; (2) Rather Explanatory; (3) Equally Pragmatic/Explanatory; (4) Rather Pragmatic; (5) Very Pragmatic.

Items	Score	Rationale
**Eligibility -** to what extent are the participants in the trial similar to those who would receive this intervention if it was part of usual care	4	Target population: Elderly with depression. Inclusion criteria are wide. No exclusion criteria will be applied in terms of setting of recruitment, severity of depression, past use of psychotropic drugs, current use of benzodiazepines, number and severity of medical comorbidities, and multiple pharmacotherapy. Diagnosis are based on clinical judgment (guided by DSM-5 criteria), as it is in usual practice. Nevertheless investigator and patient have to agree to discontinue any current antidepressant, second generation antipsychotic, or lithium.
**Recruitment** - how much extra effort is made to recruit participants over and above what that would be used in the usual care setting to engage with patients?	5	Participants will be recruited without extra efforts. They will be recruited during usual appointments and/or visits.
**Setting -** how different is the setting of the trial and the usual care setting?	4	The study is multicenter, based in more than 10 psychiatric centers of the National Health System in Italy with a University center.
**Organisation -** how different are the resources, provider expertise and the organisation of care delivery in the intervention arm of the trial and those available in usual care?	4	We will use usual staff and resources, but with some extra resources will be necessary to hire researchers and psychologists for the study.
**Flexibility (delivery) -** how different is the flexibility in how the intervention is delivered and the flexibility likely in usual care?	5	The intervention in flexible, similar to usual care.
**Flexibility (adherence) -** how different is the flexibility in how participants must adhere to the intervention and the flexibility likely in usual care?	4	No extra measures. Participants will be free to assume the intervention or drop it. But drugs will be prescribed and given to the participants during visits. This is different from usual care (patients have a prescription and go to the pharmacy to buy drugs).
**Follow-up -** how different is the intensity of measurement and follow-up of participants in the trial and the likely follow-up in usual care?	4	The primary outcome will be assessed after 1, 3 and 6 months, as it is usually done in usual practice. Six months represent a clinically sound time frame for assessing the overall tolerability of medications, including both acute, short-term and medium-long-term effects. Nevertheless, visits could be longer than usual to assess all the scales and long-term effects and adverse events could occur after 6 months.
**Primary outcome -** to what extent is the trial's primary outcome relevant to participants?	5	Primary outcome is relevant to participants and policy makers.

**Primary analysis -** to what extent are all data included in the analysis of the primary outcome?	5	The Intention to Treat (ITT) population will consist of all randomized patients. This ITT population will be used for the analysis of both primary and secondary outcomes. Missing values in rating scales will be imputed using the Last Observation Carried forward (LOCF) approach.

Figure F1. Pragmatism wheel according to the Pragmatic–explanatory continuum indicator summary-2 (PRECIS-2) tool

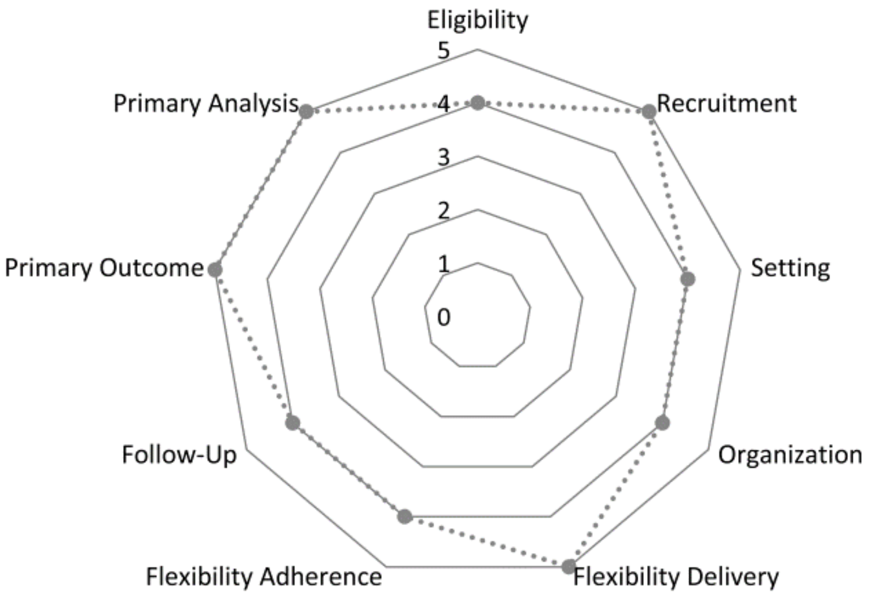

Supplement G. Antidepressants' mean dose and additional treatments

Table G1. Additional treatments prescribed during study follow-up

Additional treatments	Vortioxetine	SSRIs	p-value
Other antidepressants	12/179	12/178	1·000
Any antipsychotic	11/179	16/178	0·317
Lithium	0/179	1/178	0·316
Other mood stabilizers	12/179	15/178	0·548
Benzodiazepines	61/179	71/178	0·274
Psychological support	4/179	6/178	0·521

Table G2. Details of doses prescribed for each antidepressant

Medication	N. individuals	Minimal dose prescribed	Dose prescribed (5th percentile)	Dose prescribed (50th percentile)	Dose prescribed (95th percentile)	Maximum dose prescribed	Mean dose prescribed	SD of the mean dose prescribed	Mean treatment duration (days)
Vortioxetine	179	2·5	5·0	5·0	20·0	20·0	8·2	4·1	127·1
Sertraline	106	20·0	50·0	50·0	100·0	200·0	58·7	23·5	134·8
Citalopram	18	5·0	5·0	20·0	20·0	20·0	17·5	4·9	126·6
Escitalopram	27	0·0	8·0	10·0	20·0	20·0	11·0	4·3	140·4
Paroxetine	22	5·0	10·0	20·0	30·0	30·0	17·7	6·3	159·4
Fluoxetine	4	20·0	20·0	20·0	20·0	20·0	20·0	0·0	180·0
Fluvoxamine	1	50·0	50·0	50·0	50·0	50·0	50·0	-	121·0

Table G3. Mean antidepressants' dose prescribed during the study, expressed as the ratio between Prescribed Daily Dose and Defined Daily Dose (PDD/DDD)

	**Vortioxetine**	**SSRIs**
median (q1 - q3)	1·0 (0·5 - 1·0)	1·0 (1·0 - 1·5)
mean (SD)	1·0 (0·5)	1·3 (0·6)
(min - max)	(0·3 - 5·0)	(0·4 - 5·0)

Supplement H. List of investigators of the VESPA Study Group

**Name**	**Surname**
Eugenio	Aguglia
Andrea	Aguglia
Maria Chiara	Alessi
Gabriele	Avincola
Bianca	Bachi
Angelo	Barbato
Corrado	Barbui
Francesco	Bartoli
Gianna	Bernasconi
Andrea	Birgillito
Emanuele	Bisso
Stefano	Bonora
Angela	Calabrese
Camilla	Callegari
Tommaso	Callovini
Aurelia	Canestro
Salvo	Canonico
Chiara Alessandro	Capogrosso
Elvira	Carbone
Doriana	Carosielli
Giuseppe	Carrà
Massimo	Cartabia
Ivano	Caselli
Daniele	Cavaleri
Simone	Cavallotti
Clara	Cavallotto
Marco	Cesca
Cecilia	Chiarenza
Riccardo Matteo	Cioni
Sara	Coloccini
Marco	Cruciata
Claudia	Cumerlato
Armando	D'Agostino
Barbara	D'Avanzo
Pasquale	De Fazio
Renato	De Filippis
Manuela	De Palma
Sasha	Del Vecchio
Bianca	Della Rocca
Chiara	Di Natale
Ettore	D'Onofrio
Irene	Espa
Giulia	Fior
Marta	Gancitano
Matteo	Gari

Chiara	Gastaldon
Barbara	Giordano
Laura	Giusti
Luigi	Grassi
Pierluca	Guzzi
Marta	Ielmini
Gianmarco	Ingrosso
Celeste	Isella
Annamaria	Lax
Silvia	Mammarella
Leonardo	Marano
Federico	Marconi
Marco	Marella
Alessia	Metelli
Giulia	Michencig
Andrea	Miuli
Alessandro	Moncada
Igor	Monti
Pietro	Morello
Federico	Moretti
Marco	Morreale
Alessio	Mosca
Christian	Nasti
Michela	Nosé
Filippo	Ogheri
Margherita	Oresti
Alessandra	Ornaghi
Giovanni	Ostuzzi
Dario	Palpella
Corinna	Pancheri
Davide	Papola
Silvia	Passeri
Mauro	Pettorusso
Susanna	Piacenti
Irene	Pinucci
Valentina	Pugliese
Marianna	Purgato
Marianna	Rania
Federica	Robbi
Alessandro	Rodolico
Samantha	Romito
Barbara	Ronchi
Rita	Roncone
Valentina	Roselli
Cristina	Segura-Garcia
Maria Salvina	Signorelli
Gabriele	Simonelli
Antonella	Sociali
Enrico	Sterzi

Serena	Sturiale
Antonio	Tambelli
Mauro	Tettamanti
Beatrice	Todesco
Alice	Trabucco
Giulia	Turrini
Veronica	Villa
Federico	Wiedenmann
Luca	Zambuto
Elisa	Zanini
Chiara	Zannini
Luigi	Zerbinati
