# Supplementary material for: Tolerability of vortioxetine compared to selective serotonin reuptake inhibitors in older adults with major depressive disorder (VESPA): a randomised, assessor-blinded and statistician-blinded, multicentre, superiority trial
Source: eClinicalMedicine. 2024 Feb 15;69:102491. doi: 10.1016/j.eclinm.2024.102491 (PMC10879669; doi:10.1016/j.eclinm.2024.102491)
Supplement: Summary Italian [file mmc2.docx]

The following translations in Italian were submitted by the authors and we reproduce them as supplied. They have not been peer reviewed. Our editorial processes have only been applied to the original abstract in English, which should serve as reference for this manuscript.

**Abstract**

**Premessa.** Il disturbo depressivo maggiore (MDD) è una situazione clinica frequente e invalidante tra gli anziani. In questa popolazione, sulla base del profilo di tollerabilità, vortioxetina potrebbe rappresentare un'alternativa promettente agli inibitori selettivi della ricaptazione della serotonina (SSRI).

**Metodi.** Abbiamo condotto uno studio randomizzato di superiorità che includeva soggetti anziani con MDD. I partecipanti sono stati randomizzati al trattamento con vortioxetina o ad uno degli SSRI, scelto secondo la normale pratica clinica. Gli sperimentatori che somministravano le scale psicometriche erano in cieco rispetto alla allocazione dei partecipanti, ed il biostatistico era in cieco durante l’analisi dei dati. L'interruzione del trattamento a causa di eventi avversi dopo sei mesi era la variabile di esito primaria, per la quale avevamo ipotizzato una differenza del 12% a favore della vortioxetina rispetto al gruppo degli SSRI. Lo studio è stato registrato nell’archivio online *clinicaltrials.gov* (NCT03779789).

**Risultati.** La popolazione *“intention-to-treat”* includeva 179 individui randomizzati a vortioxetina e 178 agli SSRI. L'età media era di 73,7 anni (deviazione standard 6,1), e 264 partecipanti (69%) erano di sesso femminile. Tra coloro che assumevano vortioxetina, 78 (44%) hanno interrotto il trattamento a causa di eventi avversi a sei mesi, rispetto a 59 (33%) tra coloro che assumevano SSRI (*odds ratio* 1,56; intervallo di confidenza al 95% 1,01-2,39). Sia le analisi che hanno incluso co-variate per l’aggiustamento statistico, sia le analisi della popolazione *“per protocol”,* hanno confermato le stime puntuali a favore degli SSRI, ma senza rilevare differenze significative. Ad eccezione dell'analisi di sopravvivenza non aggiustata che favoriva gli SSRI, i risultati degli esiti secondari fornivano risultati coerenti con l'assenza di differenze sostanziali in termini di sicurezza e tollerabilità tra i due gruppi. Nel complesso, non sono emerse differenze significative per quanto riguarda i tassi di risposta, i sintomi depressivi e la qualità della vita, mentre gli SSRI apparivano favorevoli rispetto a vortioxetina in termini di performance cognitiva.

**Interpretazione.** Contrariamente a quanto ipotizzato, la vortioxetina non ha mostrato un profilo di tollerabilità migliore rispetto agli SSRI negli anziani con MDD. Inoltre, le analisi non confermano i presunti vantaggi della vortioxetina sui sintomi cognitivi correlati alla depressione evidenziati da precedenti ricerche. La potenza statistica dello studio e il disegno altamente pragmatico consentono di generalizzare i risultati alla pratica clinica del mondo reale.

**Finanziamento.** Lo studio è stato finanziato dall'Agenzia Italiana del Farmaco nell'ambito del Bando 2016 per la Ricerca Indipendente.
